# *Helicobacter pylori*-induced reactive oxygen species direct turnover of CSN-associated STAMBPL1 and augment apoptotic cell death

**DOI:** 10.1007/s00018-022-04135-2

**Published:** 2022-01-23

**Authors:** Supattra Chaithongyot, Michael Naumann

**Affiliations:** Medical Faculty, Otto von Guericke University, Institute of Experimental Internal Medicine, Leipziger Str. 44, 39120 Magdeburg, Germany

**Keywords:** Cullin-RING-ubiquitin ligase, Genotoxic stress, Ubiquitinylation

## Abstract

Deubiquitinylases (DUBs) are central regulators of the ubiquitin system involved in protein regulation and cell signalling and are important for a variety of physiological processes. Most DUBs are cysteine proteases, and few other proteases are metalloproteases of the JAB1/MPN +/MOV34 protease family (JAMM). STAM-binding protein like 1 (STAMBPL1), a member of the JAMM family, cleaves ubiquitin bonds and has a function in regulating cell survival, Tax-mediated nuclear factor kappa-light-chain-enhancer of activated B cells (NF-κB) activation and epithelial-mesenchymal transition. However, the molecular mechanism by which STAMBPL1 influences cell survival is not well defined, especially with regard to its deubiquitinylation function. Here, we show that reactive oxygen species (ROS) induced by chemotherapeutic agents or the human microbial pathogen *Helicobacter pylori* can induce cullin 1-RING ubiquitin ligase (CRL1) and 26S proteasome-dependent degradation STAMBPL1. Interestingly, STAMBPL1 has a direct interaction with the constitutive photomorphogenic 9 (COP9 or CSN) signalosome subunits CSN5 and CSN6. The interaction with the CSN is required for the stabilisation and function of the STAMBPL1 protein. In addition, STAMBPL1 deubiquitinylates the anti-apoptotic protein Survivin and thus ameliorates cell survival. In summary, our data reveal a previously unknown mechanism by which the deubiquitinylase STAMBPL1 and the E3 ligase CRL1 balance the level of Survivin degradation and thereby determine apoptotic cell death. In response to genotoxic stress, the degradation of STAMBPL1 augments apoptotic cell death. This new mechanism may be useful to develop therapeutic strategies targeting STAMBPL1 in tumours that have high STAMBPL1 and Survivin protein levels.

**Supplementary Information:**

The online version contains supplementary material available at 10.1007/s00018-022-04135-2.

## Background

Many signalling molecules and thereby cellular processes are regulated via ubiquitinylation, a reversible protein modification ultimately leading to the attachment of the 76-amino acid protein ubiquitin to the *ε*-amino groups of lysine (K) residues in target proteins, which involves a cascade of three enzymes comprising the ubiquitin-activating enzymes (E1), the ubiquitin conjugation enzymes (E2) and the ubiquitin ligases (E3) [1]. The biggest group of E3 ligases is represented by the Cullin-RING ubiquitin ligases (CRLs), which mark the proteins by K48-ubiquitinylation for subsequent degradation in the ubiquitin-proteasome system (UPS). CRLs are activated by the covalent modification of the cullin protein with the ubiquitin-like protein neuronal precursor cell-expressed developmentally down-regulated protein 8 (NEDD8). This modification results in a conformational change in the cullin C-terminus that facilitates the ubiquitinylation of substrate proteins [2]. The CSN, an evolutionarily conserved eight-subunit protein complex, negatively regulates CRLs by removing NEDD8 from activated Cullins [3].

Deubiquitinating enzymes (DUBs) oppose the action of the E3 ligases by removing ubiquitin from substrate proteins, resulting in altered protein stability or activity [4]. The human genome codes for approximately 100 DUBs, which are classified into six subclasses based on the structure of their catalytic domains and likely mechanisms of action, namely ubiquitin carboxy-terminal hydrolases (UCHs), ubiquitin-specific proteases (USPs), ovarian tumour proteases (OTUs), Jab1/Pad1/MPN domain-associated metallopeptidases (JAMMs), Machado-Joseph disease protein domain proteases, and the most recently discovered motif interacting with ubiquitin containing novel DUB family (MINDY) [5].

The deubiquitinylating enzyme STAM-binding protein like 1 (STAMBPL1), a member of the JAMM family of DUBs, comprises metalloprotease activity and cleaves K63 as well as K48 ubiquitin linkages [6, 7]. Structurally, STAMBPL1 is closely related to associated molecule with the SH3 Domain of STAM (AMSH). Both STAMBPL1 and AMSH possess an N-terminal microtubule-interacting and transport (MIT) domain, a putative nuclear localisation sequence (NLS), a C-terminal Mpr1p and Pad1p N-terminal (MPN) domain, and a JAMM motif [8]. AMSH is known as an endosome-associated DUB playing a critical role in endosomal-lysosomal sorting to facilitate the recycling of cell-surface receptors by removing K63-linked polyubiquitin on the substrates [9]. On the other hand, STAMBPL1 has a function in the regulation of cell survival [10, 11] and plays a role in the regulation of transactivator from the X-gene region (Tax)-mediated NF-κB activation [12] and epithelial-mesenchymal transition (EMT) [7]. STAMBPL1 expression is ubiquitous among a variety of human tissues [8] and shows overexpression in human cancer [7]. However, its regulation in response to chemotherapeutic agents and particularly in microbial infection by *H. pylori* is unknown.

Various agents such as chemotherapeutic drugs induce reactive oxygen species (ROS) in cells, which damage genetic information and cause mutations. ROS-inducing chemotherapeutic agents are used for the therapeutic treatment of cancer patients with the aim of causing apoptotic cell death of cancer cells [13]. The genotoxic stress response triggered by ROS clearly activates cellular signalling pathways and the activity of DUBs and E3 ligases [14]. In addition, microbial pathogens such as *H. pylori*, which colonises the gastric mucosa, initiates the production of ROS in the cells of the gastric mucosa [15] and induces cell line-dependent apoptotic cell death [16–19]. ROS regulates a number of pro- and anti-apoptotic proteins [20]. It has been described that the anti-apoptotic protein Survivin, a member of the inhibitor of apoptosis protein (IAP) family is deubiquitinylated by STAMBPL1 in renal cancer cells [21]. In contrast, E3 ubiquitin ligases S-phase kinase-associated protein 1/cullin-1/F-box (SCF) complex subunit F-box/leucine rich repeat protein 7 (FBXL7) [22, 23] and X-linked inhibitor of apoptosis (XIAP) [24] ubiquitinylate Survivin for proteasomal degradation.

In this study, we observed a ROS-dependent turnover of STAMBPL1 protein upon genotoxic stress induced by chemotherapeutics or *H. pylori* infection, which is mediated by the E3 ligase CRL1. Further, we demonstrate for the first time that STAMBPL1 physically interacts with the CSN to prevent apoptotic cell death through stabilisation of Survivin.

## Results

### *H. pylori* induces STAMBPL1 degradation

When studying anti-apoptotic proteins in cells infected with *H. pylori*, we observed that the amount of Survivin decreases during infection. A recent report showing that the amount of Survivin is regulated by STAMBPL1 [21] prompted us to investigate this mechanism in detail.

**Fig. 1:**
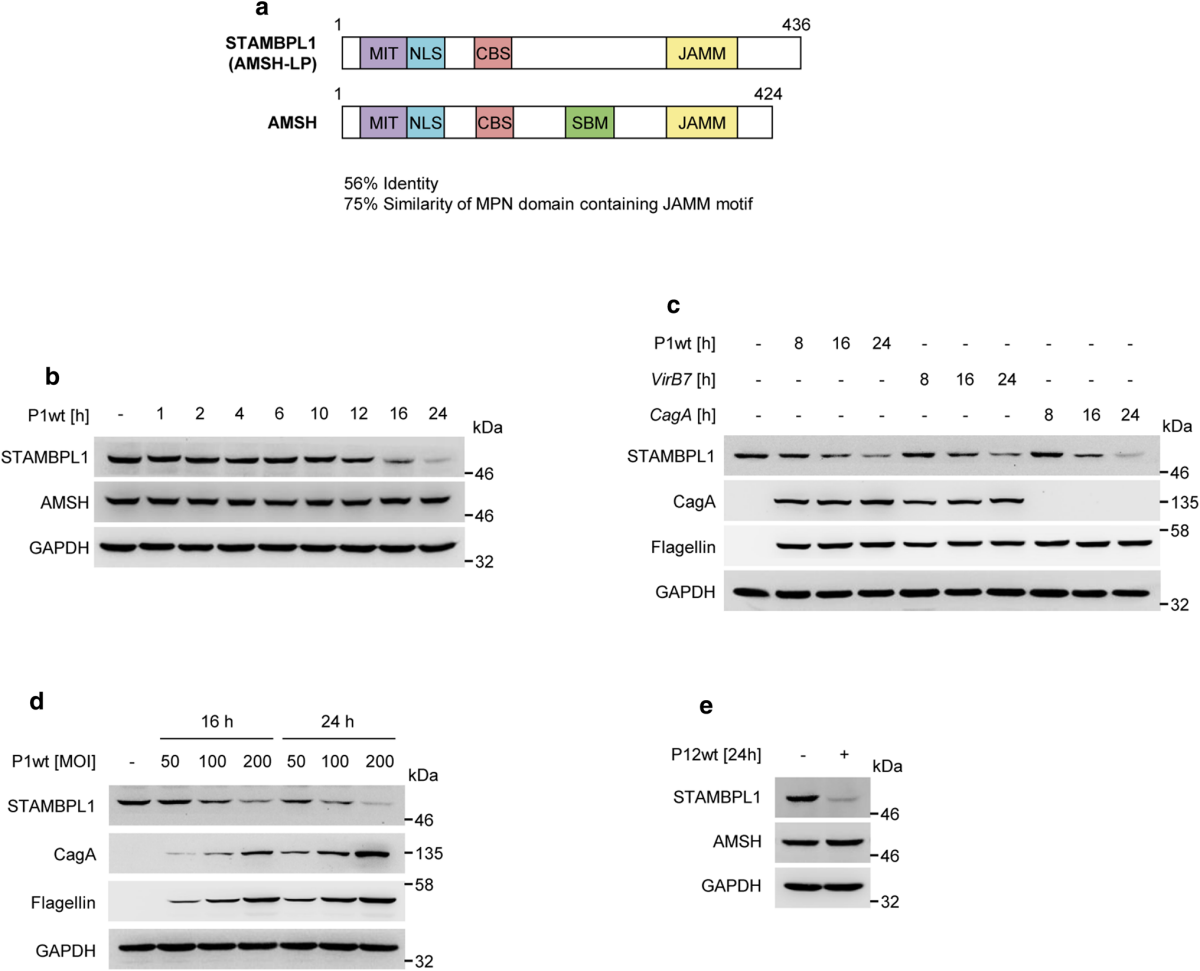
*H. pylori* infection induces STAMBPL1 protein turnover. **a** Schematic representation of the domain structure of STAMBPL1 and AMSH. MIT, microtubule-interacting and transport domain; NLS, nuclear localisation sequence; CBS, clathrin binding site; SBM, SH3-binding motif; JAMM, Jab1/Pad1/MPN domain metalloenzyme. **b** AGS cells were infected with *H. pylori* P1wt at a MOI of 100 for the indicated times and the cell lysates were subjected to immunoblot (IB) analysis. The shown blot strips for STAMBPL1 and GAPDH were selected from the same gel. **c** AGS cells were infected with different isogenic *H. pylori* strains (wt, *virB7* or *cagA*) and, **d** different MOI and then subjected to IB analysis. **e** AGS cells were infected with *H. pylori* P12wt at an MOI of 100 for 24 h. **b–e** Representative IBs for the indicated proteins from at least two independent experiments with similar results are shown

The STAMBPL1 protein comprises the domains microtubule-interacting and transport (MIT), Mpr/Pad1/N-terminal (MPN) domain, and a Jab1/MPN metalloenzyme (JAMM) domain. In addition, there is a putative nuclear localisation sequence (NLS). Structurally, STAMBPL1 is similar to associated molecule with the SH3 Domain of STAM (AMSH) with 75% identity within the JAMM domain and an overall identity of 56% (Fig. 1a, Supplementary Fig. S1a).

Studying the regulation of STAMBPL1, we observed for the first time that STAMBPL1, but not AMSH, is degraded by the genotoxic stress inducing bacterium *H. pylori* in AGS cells (Fig. 1b). Further, *H. pylori* also caused STAMBPL1 degradation in NCI-N87 and HeLa cells (Supplementary Fig. S1b–d).

Next, we investigated whether the *H. pylori* type 4 secretion system (T4SS) or the cytotoxin-associated gene A (CagA) protein are involved in *H. pylori*-induced STAMBPL1 degradation and observed that all tested strains decreased the level of STAMBPL1 protein in a time-dependent manner (Fig. 1c). In addition, an increasing multiplicity of infection (MOI) decreased the STAMBPL1 protein level congruently (Fig. 1d). Similar data were received when we studied AGS cells infected with the *H. pylori* strain P12 (Fig. 1e). The subsequent experiments were performed with the P1 strain only.

### Genotoxic stress-induced STAMBPL1 degradation is ROS-dependent

**Fig. 2:**
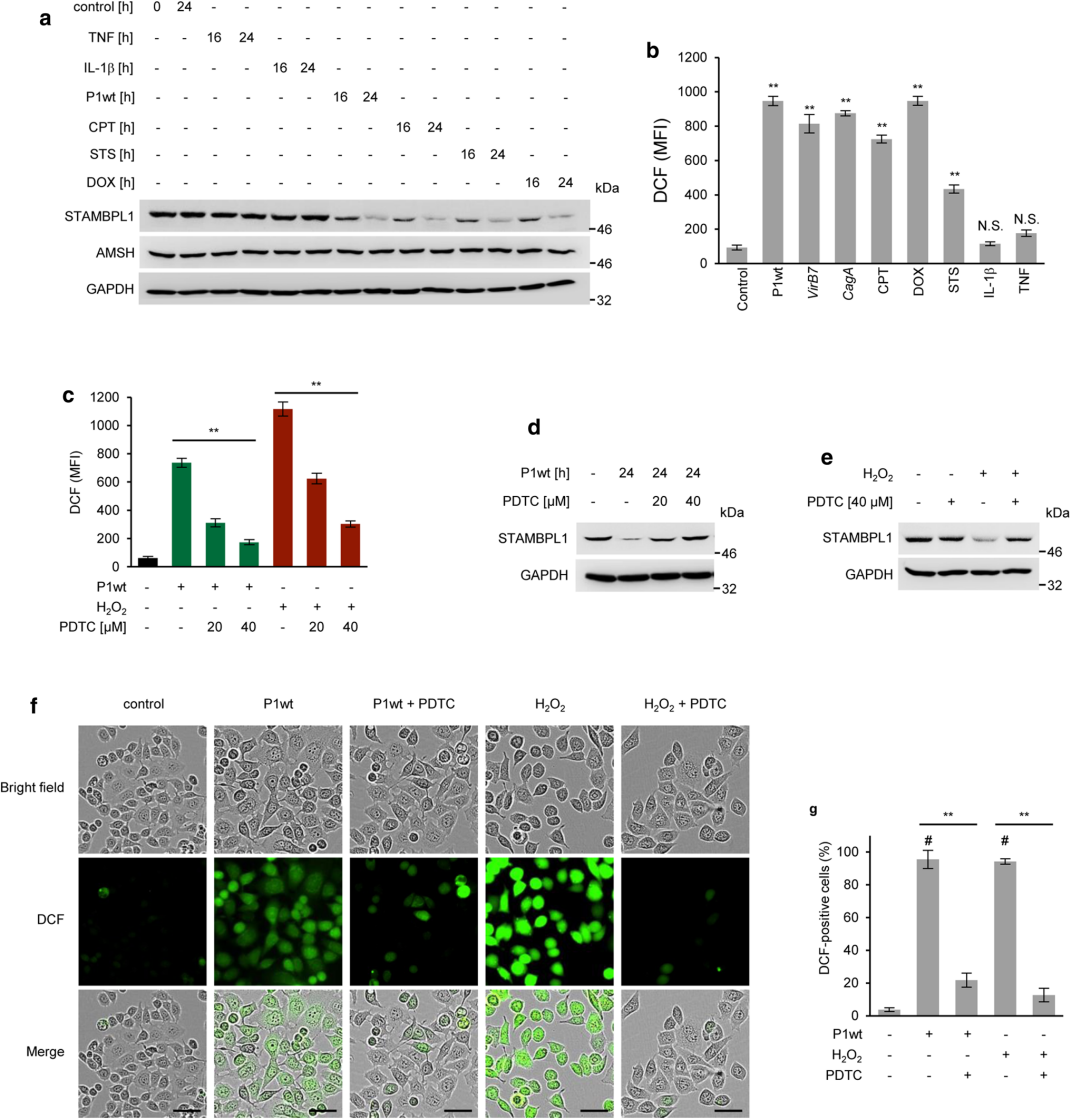
Genotoxic stress-induced STAMBPL1degradation is ROS-dependent. **a** AGS cells were infected with P1wt at a MOI of 100 or treated with chemotherapeutic agents [camptothecin (CPT), staurosporine (STS) or doxorubicin (DOX)], or cytokines (TNF or IL-1*β*) for the times indicated. The STAMBPL1 and AMSH proteins were analysed by IB. The shown blot strips for STAMBPL1 and GAPDH were selected from the same gel. **b** For ROS measurement, cells were treated with different stimuli for 4 h and then analysed with a cell-based 2ʹ,7ʹ-dichlorodihydrofluorescein (H2DCF-DA) assay assessed by flow cytometry (**p ≤ 0.01; N.S., not statistically significant; *n* = 3). **c** AGS cells were treated with pyrrolidine dithiocarbamate (PDTC) 1 h before infection. Subsequently, cells were infected with *H. pylori* at MOI 100 or treated with 500 µM of hydrogen peroxide (H_2_O_2_) for 4 h. ROS production was analysed by flow cytometry (**p ≤ 0.01; *n* = 2). **d**–**e** STAMBPL1 protein levels were analysed by IB. **f** Representative images of ROS generation in AGS cells after *H. pylori* infection or H_2_O_2_ stimulation for 4 h were acquired using IncuCyte^®^ S3 Live Cell Analysis System. Scale bars, 20 μm. PDTC was used at a final concentration of 40 μM. **g** The percentage of DFC-positive cells is shown [^#^significantly different from the control group, **Significantly different from the treatment group (*p *≤ 0.01; *n *= 2)]. **b**–**e** Representative IBs for the indicated proteins from at least two independent experiments with similar results are shown

We investigated whether genotoxic stress induced by *H. pylori* or chemotherapeutic agents [camptothecin (CPT), doxorubicin (DOX), and staurosporine (STS)] could regulate the STAMBPL1 abundance. Interestingly, in cells treated with the aforementioned chemotherapeutics, the effect on STAMBPL1 degradation was similar to that observed in the presence of *H. pylori,* whereas the treatment with cytokines had no impact (Fig. 2a). In contrast, AMSH showed no turnover (Fig. 2a).

*H. pylori* is known to induce ROS in human gastric epithelial cells [25] and most genotoxic drugs generate ROS in cancer cells [26]. Thus, prompting us to examine if ROS could be involved in the *H. pylori*-induced loss of STAMBPL1. Here, a redox-sensitive fluorescent dye 2′-7′-Dichlorodihydrofluorescein diacetate (H2-DCFDA) was used to measure ROS production. H2-DCFDA is cleaved by intracellular esterases, resulting in a charged H2-DCF molecule, which is then oxidized by ROS to produce the fluorescent molecule, DCF. *H. pylori* and chemotherapeutic agents dramatically enhanced DCF fluorescence levels, whereas no significant increase in DCF fluorescence was detected in IL-1*β* or TNF-treated cells (Fig. 2b).

Further, we pre-treated *H. pylori*-infected AGS cells with pyrrolidine dithiocarbamate (PDTC), a ROS scavenger, and determined the fluorescent intensity of DCF assessed by flow cytometry. We observed that increasing amounts of PDTC significantly resulted in a dose-dependent decrease in DCF fluorescence in *H. pylori* infected- or hydrogen peroxide-treated cells (Fig. 2c). In concordance with these data, pre-treatment of *H. pylori*-infected AGS cells with PDTC inhibited the degradation of STAMBPL1 (Fig. 2d), indicating that *H. pylori*-directed ROS causes STAMBPL1 degradation. Consistently, treatment of cells with hydrogen peroxide also induced a loss of STAMBPL1 while PDTC markedly suppressed hydrogen peroxide-induced STAMBPL1 degradation (Fig. 2e). To further corroborate our findings, we assessed by Incucyte^®^ Live-Cell Analysis the generation of ROS in AGS cells. We observed an increased ROS generation in AGS cells following *H. pylori* infection or hydrogen peroxide treatment, which was diminished by PDTC (Fig. 2f, g). Collectively, we have shown that ROS generation by *H. pylori* or treatment with chemotherapeutics led to the degradation of STAMBPL1 in gastric epithelial cells.

### CRL1-, and 26S proteasome-dependent degradation of STAMBPL1

**Fig. 3:**
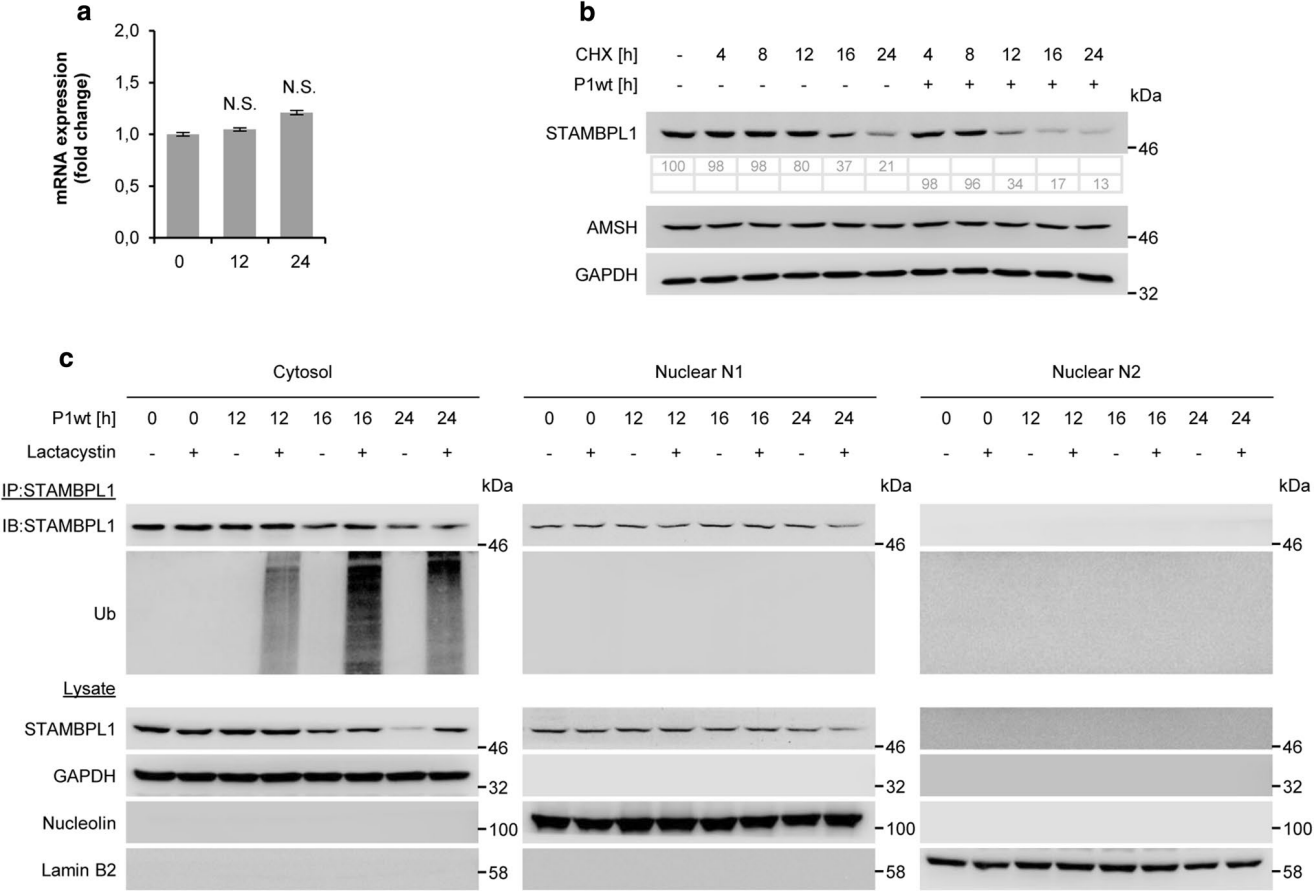
26S proteasome-dependent degradation of STAMBPL1. **a** Total RNA was isolated after *H. pylori* infection at the indicated time points and changes in STAMBPL1 transcript expression were examined by quantitative PCR (N.S., not statistically significant; *n *= 3). **b** AGS cells were treated with CHX at the indicated time points, or 30 min prior to *H. pylori* infection at times indicated. Quantification of band intensities was performed using ImageJ software. The shown blot strips for STAMBPL1 and GAPDH were selected from the same gel. **c** Cells were infected with *H. pylori* at the indicated time points and subjected to subcellular fractionation. GAPDH, nucleolin and lamin B2 served as controls for purity of subcellular fractions and equal amount of protein loading. STAMBPL1 was immunoprecipitated (IP) from each fraction and the IP subjected to IB analysis. Lactacystin at a final concentration of 10 µM was added 4 h before harvesting. **b**,** c** Representative IBs for the indicated proteins from at least two independent experiments with similar results are shown

To assess whether the *H. pylori*-induced down-regulation of STAMBPL1 was due to a transcriptional mechanism, we initially analysed the mRNA expression and observed that *H. pylori* did not inhibit STAMBPL1 mRNA expression (Fig. 3a). Next, we investigated STAMBPL1 protein stability in the presence of cycloheximide (CHX) and found that *H. pylori* infection accelerated the protein turnover of STAMBPL1 (Fig. 3b). In contrast, AMSH showed no turnover during CHX treatment (Fig. 3b).

Since the UPS is involved in the degradation of the majority of proteins, we therefore examined the 26S proteasome participation in the turnover of STAMBPL1 using the proteasome inhibitor Lactacystin in *H. pylori* infected cells. Here, we observed that the STAMBPL1 protein was stabilised. Consistently, we found an accumulation of K48-ubiquitinylated STAMBPL1 protein in the samples which were treated with Lactacystin (Fig. 3c). Although, STAMBPL1 is localised also in the nucleus, we found that *H. pylori* promoted STAMBPL1 K48-ubiquitinylation exclusively in the cytoplasm. These findings demonstrate that the downregulation of STAMBPL1 by *H. pylori* was accomplished via the ubiquitin-proteasome degradation pathway.

**Fig. 4:**
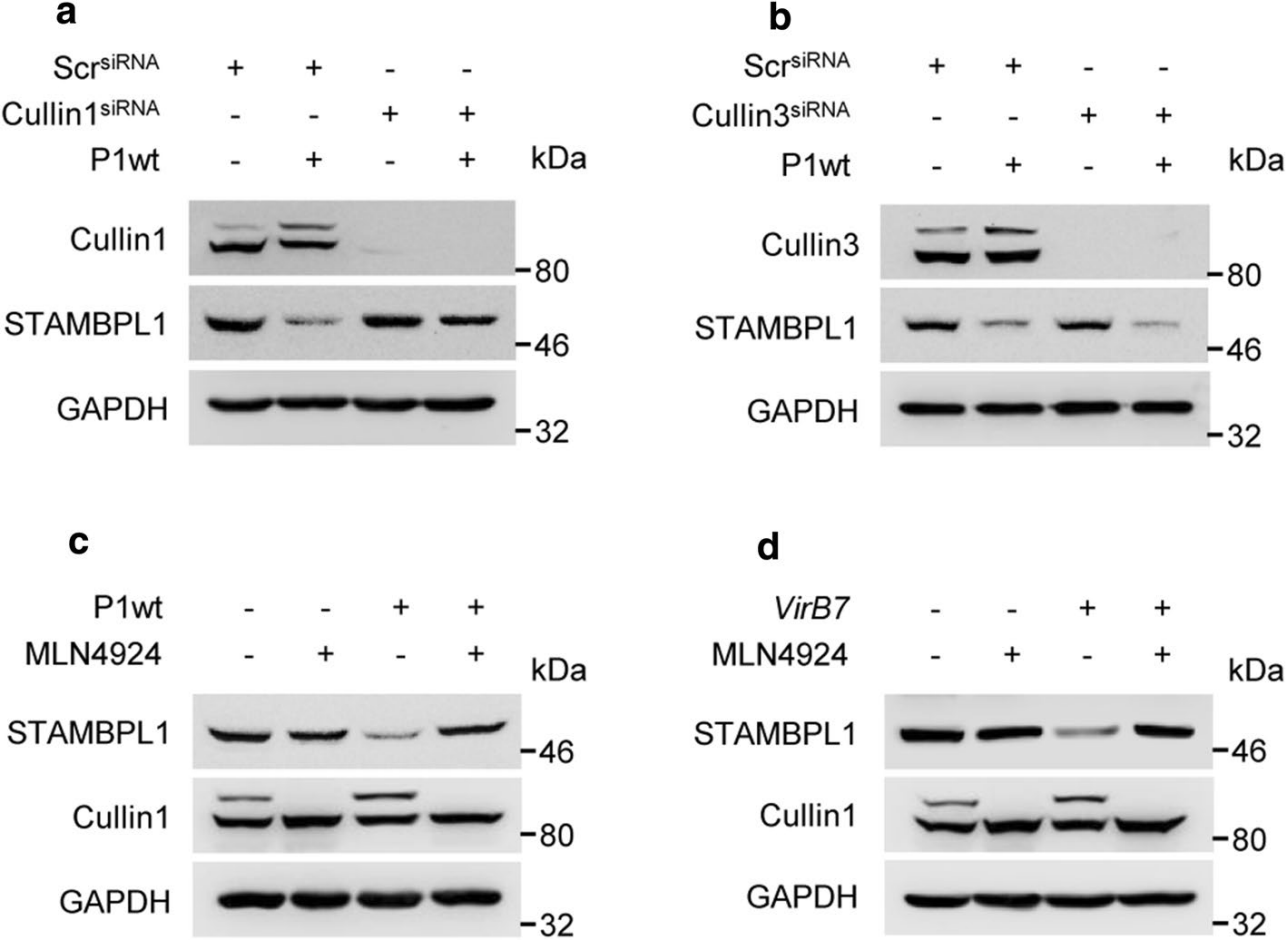
CRL1-dependent degradation of STAMBPL1. AGS cells were transfected with (**a**) cullin 1 or (**b**) cullin 3 siRNA for 48 h, followed by infection with *H. pylori* for a further 24 h. STAMBPL1 protein level was analysed by IB. **c** Cells were treated with MLN4924 at a final concentration of 1 µM and infected with *H. pylori* P1wt or (**d**) v*irB7*, followed by IB analysis of STAMBPL1. **a**–**d** Representative IBs for the indicated proteins from at least two independent experiments with similar results are shown

E3 ubiquitin ligases targeting proteins for proteasomal degradation. Within the largest family of E3 ubiquitin ligases, the cullin-RING ubiquitin ligases (CRLs) CRL1 and CRL3 have been shown to be involved in the regulation of the oxidative stress response [27, 28]. To identify the CRLs involved in STAMBPL1 regulation, we knocked down CRL1 and CRL3. We observed that loss of CUL1 stabilised STAMBPL1 protein within *H. pylori* infection. This suggests that CRL1 is involved in *H. pylori*-induced ubiquitinylation of STAMBPL1, but CRL3 is not (Fig. 4a, b).

The activity of CRLs is regulated by covalent attachment with the ubiquitin-like protein NEDD8, which requires the activity of the NEDD8-activating enzyme, whose activity can be blocked by the small molecule MLN4924. Consequentially, we observed that the treatment with MLN4924 protected against *H. pylori*-induced STAMBPL1 loss in both P1wt (Fig. 4c, Supplementary Fig. S2a) and the *virB7* mutant (Fig. 4d, Supplementary Fig. S2b).

### STAMBPL1 is a novel CSN-associated DUB

**Fig. 5:**
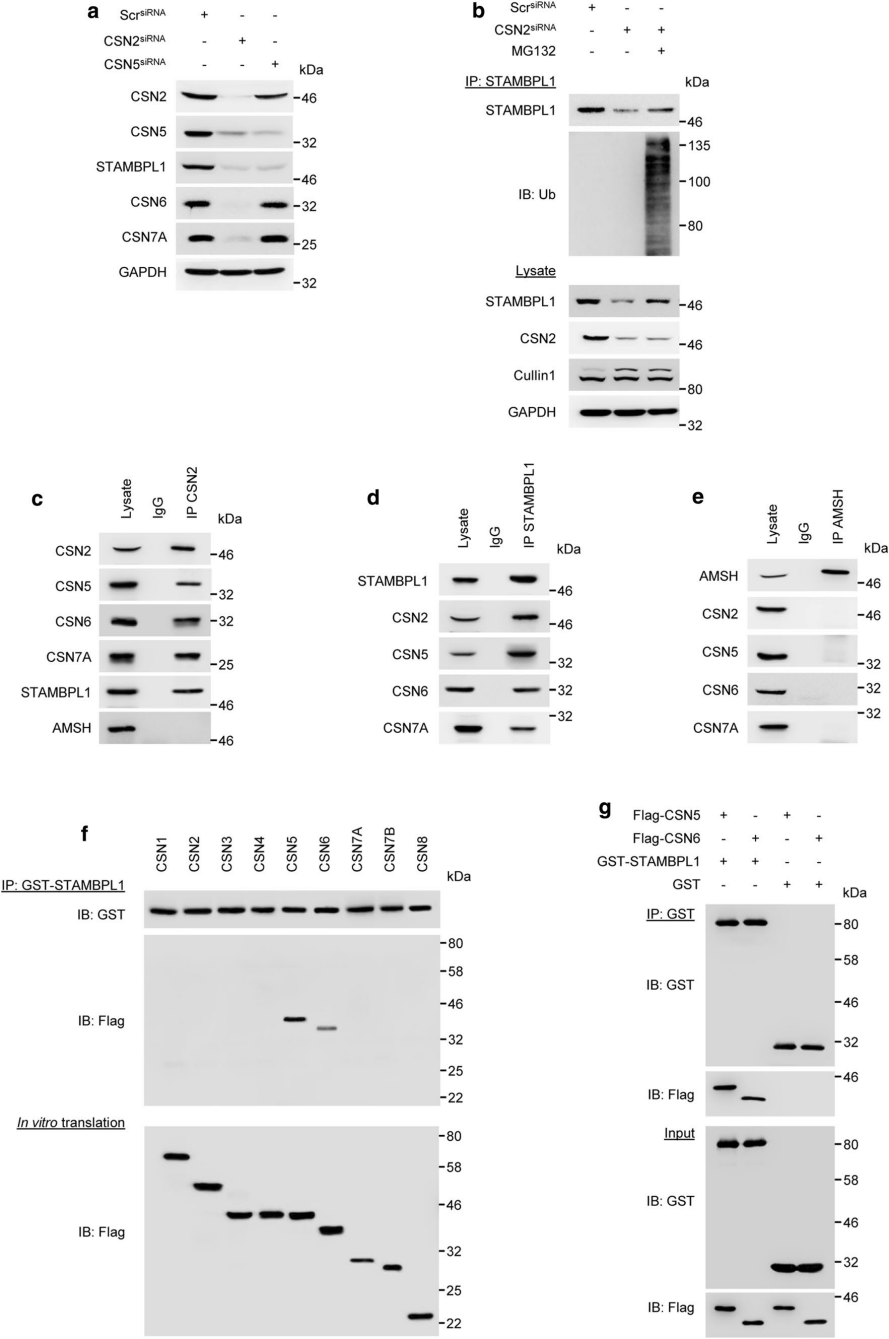
STAMBPL1 interacts with the CSN. **a** AGS cells were transfected with siRNA against CSN2 or CSN5 for 48 h, followed by IB analysis. **b** AGS cells depleted of CSN2 were treated with MG132 at a final concentration of 20 µM 4 h prior to harvest and subjected to IP with an anti-STAMBPL1 antibody. Ubiquitinylation of STAMBPL1 was analysed by IB. **c**–**e** IPs of CSN2, STAMBPL1, and AMSH from AGS cell lysates which were analysed by IB. **f**, **g** In vitro translation and binding assay of Flag-CSN subunits and recombinant GST-STAMBPL1. Equimolar amounts of in vitro-translated Flag-CSN and the recombinant GST-STAMBPL1 were incubated at 37 °C for 1 h followed by IP of STAMBPL1 using an anti-GST antibody. **g** In addition to Flag-CSN 5 and 6 subunits and recombinant GST-STAMBPL1, recombinant GST-protein was used as a negative control. **a**–**g** Representative IBs for the indicated proteins from at least two independent experiments with similar results are shown

The multifunctional protein CSN complex is composed of eight subunits (CSN1-8) and exerts deneddylase activity executed by the catalytic subunit CSN5 [29, 30]. Depletion of CSN2 affect the protein stability of other CSN subunits [31, 32]. Since the CSN regulates CRLs by removing NEDD8, we investigated whether CSN participates in STAMBPL1 stability. The knock down of CSN2 by siRNA led to a loss of the STAMBPL1 protein and loss of other CSN subunits, whereas the CSN5 knockdown led only to the loss of STAMBPL1 (Fig. 5a). In addition, we observed in CSN2 knockdown cells treated with the proteasome inhibitor MG132, a significant accumulation of ubiquitinylated STAMBPL1 (Fig. 5b), suggesting that a disruption of CSN function enhanced the degradation of STAMBPL1 by CRL1.

It is well established that the CSN represents a signalling platform and collaborates with a number of other proteins including DUBs [33]. Interestingly, we found that STAMBPL1, but not AMSH was co-immunoprecipitated with the CSN complex and vice versa (Fig. 5c–e). To elucidate the direct binding of STAMBPL1 to the CSN further, single CSN subunits were in vitro translated, incubated with recombinant GST-STAMBPL1 and subjected to an IP using an anti-GST antibody. We detected direct physical interaction between STAMBPL1 and the CSN subunits CSN5 and CSN6 (Fig. 5f, g). No interaction between recombinant GST and the CSNs was observed (Fig. 5g). Furthermore, we found that USP15 directly interacts with CSN subunit 7A (Supplementary Fig. S3), which was also shown in a co-immunoprecipitation from cell lysates [34].

### STAMBPL1 stabilises the anti-apoptotic protein Survivin by deubiquitinylation

**Fig. 6:**
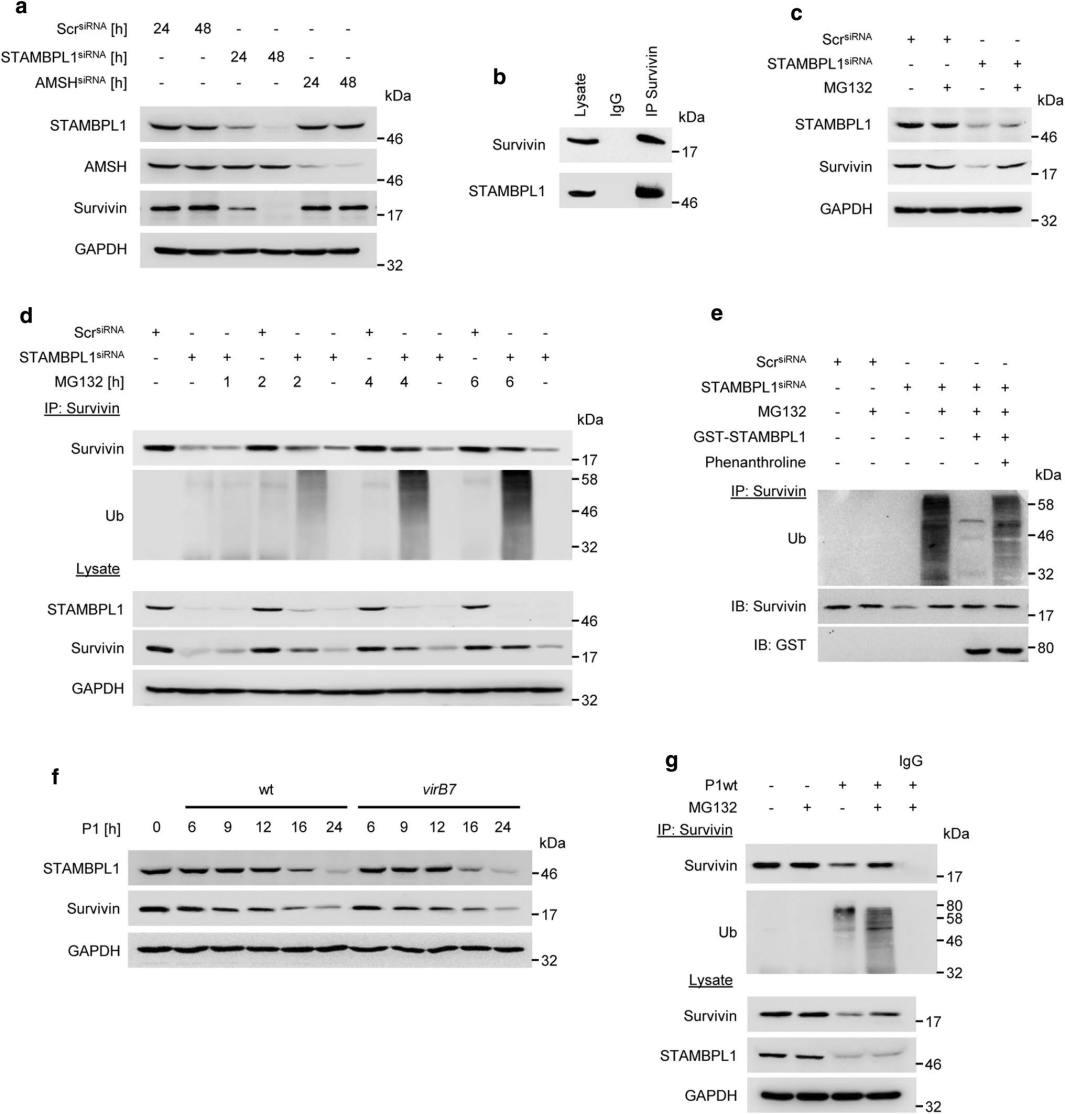
STAMBPL1 stabilises the anti-apoptotic protein Survivin by deubiquitinylation. **a** AGS cells were transfected with siRNAs against STAMBPL1 or AMSH at the indicated time points, followed by IB analysis. **b** IP of Survivin from AGS cell lysates. **c** Cells were transfected with STAMBPL1 siRNA for 24 h and treated with MG132 for a further 4 h before harvesting. **d** IP of Survivin after transient knockdown of STAMBPL1 followed by IB analysis of Survivin ubiquitinylation. **e** In vitro DUB assay of a Survivin IP from STAMBPL1 depleted cells incubated for 2 h with recombinant GST-STAMBPL1 in the presence or absence of phenanthroline. **f** Cells were infected with either P1wt or *virB7* at the indicated time points, followed by IB analysis of Survivin. **g** IP of Survivin from *H. pylori*-infected cells. MG132 was added to the culture media 4 h before harvest. **a**–**g** Representative IBs for the indicated proteins from at least two independent experiments with similar results are shown

It has been described that STAMBPL1 stabilises the anti-apoptotic regulator Survivin in renal cancer cells [21]. We also observed that Survivin protein abundance was found to be significantly decreased in STAMBPL1-depleted gastric epithelial cells, whereas AMSH had no impact on Survivin (Fig. 6a). Further, STAMBPL1 co-precipitated with Survivin in an IP (Fig. 6b). The STAMBPL1-dependent Survivin degradation is proteasome-dependent (Fig. 6c) and we observed an accumulation of ubiquitinylated Survivin in STAMBPL1-depleted and MG132 treated cells (Fig. 6d). In order to test the hypothesis that STAMBPL1 deubiquitinylates Survivin, we performed an in vitro DUB assay. STAMBPL1 effectively hydrolysed polyubiquitin chains on Survivin in vitro while phenanthroline, a metalloprotease inhibitor, completely inhibited the cleavage of ubiquitin on Survivin (Fig. 6e). This data provide evidence that the DUB activity of STAMBPL1 regulates the stability of Survivin.

Along these lines, it is conceivable that STAMBPL1 down-regulation by *H. pylori* infection causes the degradation of Survivin. We observed in agreement with the *H. pylori*-induced degradation of STAMBPL1 a decrease in the Survivin protein level (Fig. 6f). Moreover, we detected an enhanced polyubiquitinylation of Survivin upon *H. pylori* infected and MG132 treated cells (Fig. 6g) suggesting that STAMBPL1 deubiquitinylates Survivin and sustains Survivin stability in AGS cells.

### *H. pylori*-induced degradation of STAMBPL1 promotes apoptotic cell death

**Fig. 7:**
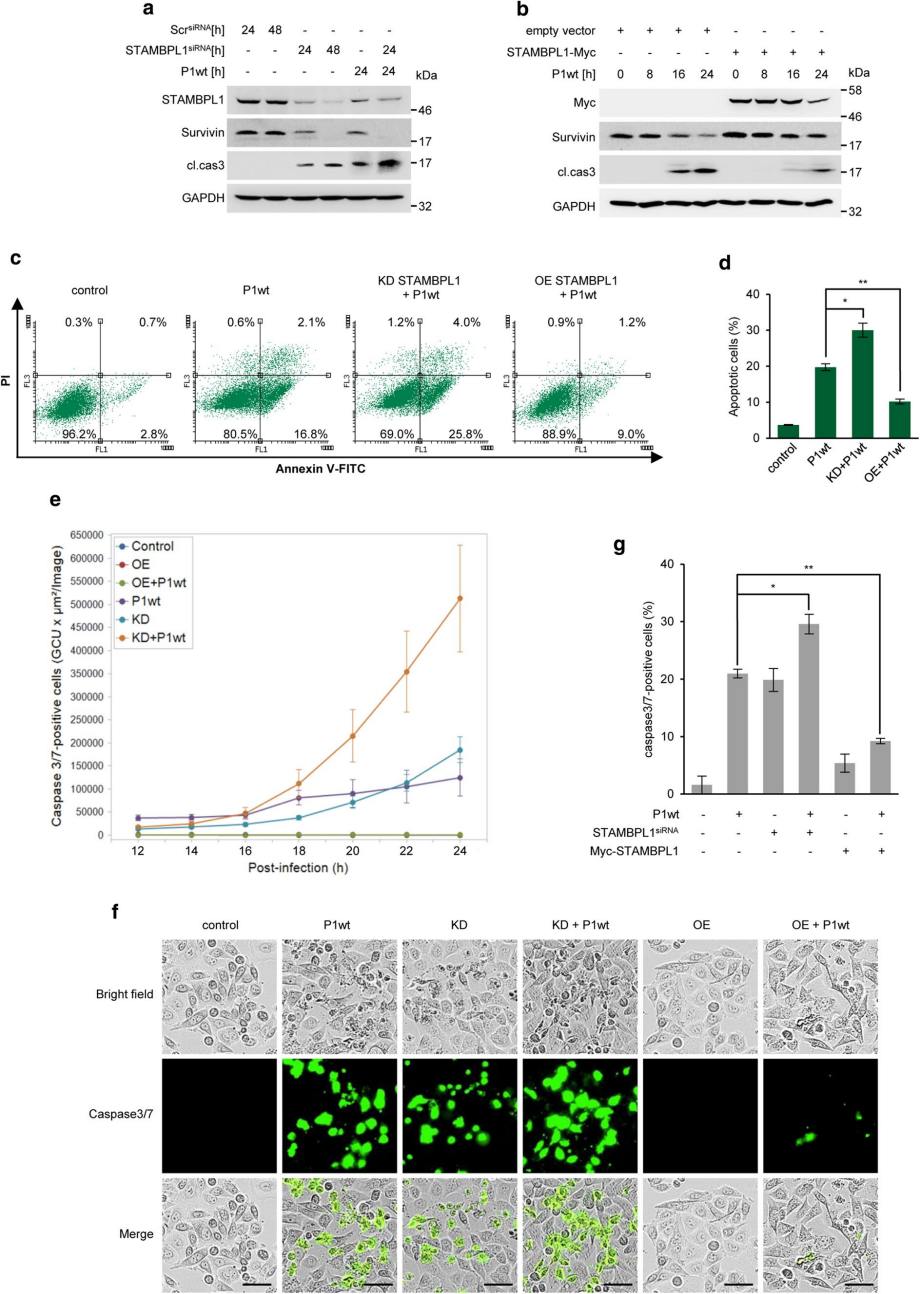
*H. pylori*-induced degradation of STAMBPL1 promotes apoptotic cell death. **a** AGS cells were transfected with siRNA against STAMBPL1 for 24 h and then infected with *H. pylori* for 24 h. Survivin and caspase-3 cleavage were analysed by IB. **b** AGS cells were transfected with either empty vector control (pCMV) or STAMBPL1 cDNA for 24 h prior to *H. pylori* infection for the indicated time points. **c** AGS cells were transfected with either STAMBPL1 siRNA (KD = knockdown cells) or STAMBPL1 cDNA (OE = overexpression). 24 h after transfection, cells were infected with *H. pylori* for the indicated periods and subsequently stained with Annexin V/PI. Apoptotic cell death was analysed by flow cytometry. **d** Shown is the percentage of total apoptotic cells (**p ≤ 0.01; *n *= 2). **e** Caspase-3/7 activation in AGS cells infected with *H. pylori* was analysed in real time using the IncuCyte^®^ S3 Live Cell Analysis System. **f** Representative images after 24 h and (**g**) the percentage of caspase-3/7-positive cells are shown. Scale bars, 20 μm. **a**, **b** Representative IBs for the indicated proteins from at least two independent experiments with similar results are shown

Given that the STAMBPL1 deubiquitinylase activity stabilises Survivin, a crucial regulator of apoptosis at the level of effector caspases, we hypothesized that STAMBPL1 affects apoptotic cell death. We observed that a knockdown of STAMBPL1 in non-stimulated cells induced caspase-3 cleavage, which was raised by *H. pylori*-infected cells and even more pronounced in cells which were additionally transfected with siRNA against STAMBPL1 (Fig. 7a, Supplementary Fig. S4). This effect is due to the fact, that *H. pylori* infection induces a number of factors that might contribute to the regulation of apoptotic cell death [19].

The impact of STAMBPL1 on apoptotic cell death in *H. pylori*-infected cells was further analysed by overexpression of a Myc-tagged STAMBPL1. Overexpression of STAMBPL1 in *H. pylori*-infected cells notably promoted the stability of Survivin and diminished the caspase-3 cleavage (Fig. 7b).

Annexin V-FITC/Propidium Iodide staining assessed by flow cytometry corroborated the previous data and showed that apoptotic cell death in *H. pylori* infection was even more pronounced when cells were transfected with STAMBPL1 siRNA, whereas overexpression of STAMBPL1 markedly reduced apoptotic cell death (Fig. 7c, d).

The suppressive effect of STAMBPL1 on apoptotic cell death in *H. pylori* infection was also observed when we studied apoptotic cell death by Incucyte^®^ Live-Cell Analysis. Using caspase-3/7 Incucyte^®^ reagent, a non-fluorescent membrane-permeable substrate containing a cleavage sequence (DEVD), we monitored the cleavage by activated caspase-3/7 and the release of the green DNA-binding fluorescent dye. Caspases-3/7 cleavage increased continuously in a time-dependent manner in *H. pylori* infection (Fig. 7e). Representative images of *H. pylori* infected AGS cells after 24 h are shown (Fig. 7f). Furthermore, the caspases-3/7 activation was significantly increased when STAMBPL1 was depleted, whereas it was considerably lowered in STAMBPL1 overexpressing cells (Fig. 7f, g).

## Discussion

In cells, ROS act as signalling molecules to regulate biological processes, whereas excessive ROS impairs proteins and other biomolecules, ultimately leading to cell death [35]. Chemotherapeutic drugs induce genotoxic stress and ROS, which is a hallmark in cancer therapy. Further, the bacterium *H. pylori* induces ROS in gastric epithelial cells, which leads to apoptotic cell death [19]. Our results showed the accumulation of ROS after treatment with chemotherapeutic agents or *H. pylori* infection and a ROS-dependent decline of the STAMBPL1 protein amount (Fig. 2c–e). Cytokines (IL-1*β* or TNF) do not induce ROS (Fig. 2b) and therefore we observed no cytokine-dependent effect on the turnover of STAMBPL1 protein (Fig. 2a).

It is known that cellular ROS leads to an increased turnover of proteins [36] involving the activity of E3 ubiquitin ligases, e.g. CRL1 and 3, which mark the proteins by K48-ubiquitinylation for subsequent degradation in the UPS [28]. In this study, we identified E3 ligase CRL1 executing the ubiquitinylation of STAMBPL1 in *H. pylori* infection (Fig. 4a). Interestingly, only STAMBPL1 localised in the cytosol was degraded, whereas the nuclear population of the protein was unaffected (Fig. 3c). This is consistent with other studies showing ROS-dependent turnover of cytosolic proteins [37]. There are a number of substrate binding proteins described [beta-transducin repeats-containing protein (*β*-TrCP), cell division control protein 4 (CDC4), S-phase kinase-associated protein 2 (SKP2), Cyclin F] which could be assembled in the CRL1 complex [38]. However, the identification of the substrate binding protein, which recognises STAMBPL1 demands further investigations.

The CSN exerts deneddylase activity and controls the CRLs by mediating deneddylation and subsequent assembly/disassembly of CRL complexes [33]. Accordingly, disruption of CSN function enhanced STAMBPL1 degradation by the E3 ligase CRL1 (Fig. 5a, b). The CSN functions as a signalling platform and integrates the activity of several proteins including DUBs [33, 39]. CSN-associated DUBs USP15 and USP48 contribute to NF-κB regulation [40, 41] and cylindromatosis (CYLD) is involved in hepatic steatosis [39]. Here, we identified STAMBPL1 as a novel CSN-associated DUB (Fig. 5c). Direct physical interaction was observed with CSN subunits CSN5 and CSN6 (Fig. 5f, g). The MPN domain of CSN5 has been previously demonstrated to mediate the binding of the CSN complex to several proteins e.g. cell cycle inhibitor p27 [42], DNA topoisomerase II*α* [43], macrophage migration inhibitory factor (MIF) [44], and LIM as well as SH3 protein 1 (LASP1) [45]. Here, the MPN domain of CSN5, but not the JAMM motif, is required for the interaction of CSN5 with binding partner proteins. In our study, we observed the interaction of STAMBPL1 with both MPN domain proteins, CSN5 and CSN6. Accordingly, although STAMBPL1 contains a JAMM motif, it is likely that instead of the JAMM motif, the MPN domain may facilitate the interaction between CSN proteins CSN5 and CSN6 with STAMBPL1.

In response to ROS, many proteins have been shown to be K48-ubiquitinylated and readily degraded by the 26S proteasome [46]. Therefore, it is likely that ROS directly leads to CRL1-dependent ubiquitinylation of CSN-associated STAMBPL1 and marks it for degradation. In addition, protein modifications that regulate the interaction between STAMBPL1 and the CSN, and provide access to the ROS-dependent turnover of STAMBPL1 might be involved.

A variety of signalling molecules such as inhibitor of IκB kinases (IKKs), c-Jun N-terminal kinase (JNK), mitogen-activated protein (MAP) kinases (p38), transcription factors [activator protein 1 (AP-1) and NF-κB] are initiated and regulated by the T4SS, but independent of CagA [47]. However, STAMBPL1 is regulated by *H. pylori* in a T4SS-independent manner (Fig. 1c), similar to epithelial growth factor receptor (EGFR) [48], extracellular-signal regulated kinase (ERK) and the S6 ribosomal protein [49].

*H. pylori* activates the classical and alternative NF-κB pathways, which are involved in the regulation of pro-, and anti-apoptotic genes including the inhibitor of apoptosis proteins (IAPs) [50, 51]. In addition, expression of the NF-κB induced deubiquitinylase A20 bifunctionally terminates NF-κB activation, but also negatively regulates apoptotic cell death [52]. The IAP protein Survivin has been described as negative regulator of caspases in apoptosis by blocking caspase activation [53]. STAMBPL1 was recently reported to regulate Survivin abundance in renal carcinoma [54]. Similarly, we show that STAMBPL1 stabilises the Survivin protein level (Fig. 6a and d). The molecular basis behind our observations could be explained by STAMBPL1-dependent deubiquitinylation of Survivin (Fig. 6e). The loss of STAMBPL1 is accompanied by loss of Survivin in *H. pylori*-infected cells (Fig. 6f). In prostate cancer, STAMBPL1 did not affect the stability of Survivin protein [10], which might differ because of genetic alterations in this cancer cells.

Due to the known role of Survivin in apoptotic cell death [54, 55], we hypothesized that STAMBPL1-dependent Survivin degradation is crucial for the apoptotic cell death during *H. pylori* infection. Survivin forms a complex with hepatitis B virus X-interacting protein (HBXIP) to prevent the activation of caspase cascade [56]. Survivin also blocks the release of Apaf1 from the mitochondria or prevents the second mitochondrial-derived activator of caspases (Smac) from interacting with other IAPs [57], thus, inhibiting the apoptotic pathway. STAMBPL1 knockdown in non-stimulated cells caused moderate caspase-3 cleavage, which was considerably elevated in *H. pylori*-infected cells (Fig. 7a). This is due to the destabilisation of Survivin because overexpression of STAMBPL1 in *H. pylori*-infected cells resulted in stabilisation of the Survivin protein and decreased caspase-3 cleavage (Fig. 7b). This supports our suggestion that STAMBPL1 prevents caspase-3 cleavage in *H. pylori* infection. Further, evidence for an anti-apoptotic effect of STAMBPL1 was provided by decreased numbers of apoptotic cells (Fig. 7c and d) and detection of less caspase-3 cleavage (Fig. 7e–g) in cells that overexpress STAMBPL1. Our findings strongly suggest that STAMBPL1 degradation is involved in apoptotic cell death observed in *H. pylori* infection. In addition, with increasing stages of gastric cancer (stage I-IV), STAMBPL1 protein expression was considerably upregulated [11], which could explain the emergence of apoptotic resistance in gastric carcinogenesis.

**Fig. 8:**
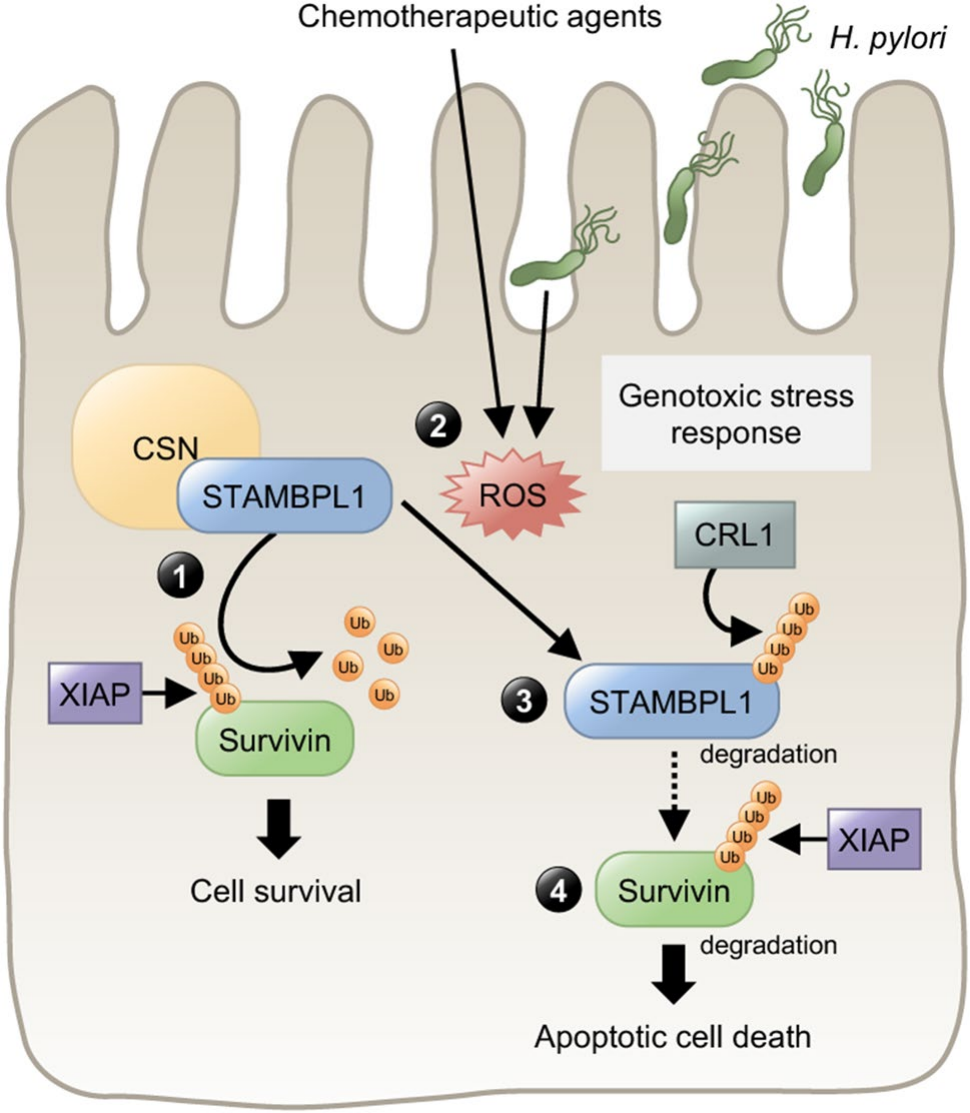
Schematic diagram summarizing the major findings of this study. **1** STAMBPL1 physically interacts with the CSN complex to enhance cell survival by stabilising the anti-apoptotic protein Survivin by counteracting XIAP-dependent degradation of Survivin. **2** The ROS-dependent turnover of the STAMBPL1 protein during genotoxic stress (chemotherapeutic agents or *H. pylori*) (**3**) is mediated by E3 ligase CRL1. **4** Degradation of STAMBPL1 also leads to degradation of Survivin (in the absence of STAMBPL1s protective function) and apoptotic cell death

In conclusion, we elucidated for the first time, that the CSN interacts with STAMBPL1 and its DUB activity stabilises the anti-apoptotic protein Survivin. Chemotherapeutic agents and *H. pylori* infection induced ROS leads to the degradation of STAMBPL1 by the E3 ligase CRL1 and augments apoptotic cell death (Fig. 8). Herein, our data provide novel insights into the pathophysiology of *H. pylori* infection and might be useful to develop therapeutic strategies targeting STAMBPL1 in cancer.

## Materials and methods

### Cell culture and bacteria

Human gastric carcinoma AGS and NCI-N87 cells and human cervical carcinoma HeLa cells (ATCC) were cultured in RPMI 1640 medium (Gibco^®^/Life Technologies) supplemented with 10% heat-in-activated foetal calf serum (FCS) (Gibco^®^/Life Technologies) in a humidified incubator at 37 °C with 5% CO_2_ and passaged once every 2–3 days.

*H. pylori* strain P1wt (wildtype) and isogenic mutants P1*cagA* (CagA-deficient) and P1*virB7* (T4SS-deficient) as well as strain P12 were grown on agar plates containing 10% horse serum, 5 µg/ml trimethoprim, 1 µg/ml nystatin, and 10 µg/ml vancomycin (AppliChem) under microaerophilic conditions at 37 °C for three days. For the P1*cagA* and P1*virB7* strains, the agar plates were supplemented with chloramphenicol (Sigma). Bacteria were replated and cultured for another two days before use.

### Transfection of siRNAs and plasmids

Cells (0.4 × 10^6^ per 60 mm or 0.8 × 10^6^ per 100 mm culture dish) were transfected with siRNAs against STAMBPL1, AMSH, Cul3 (Eurofins Genomics), Cul1 (Dharmacon), CSN2, and CSN5 (Thermo Fisher Scientific) using METAFECTENE^®^ PRO transfection reagent (Biontex) according to the manufacturer’s protocol. The siRNAs were used at a final concentration of 50 nM for STAMBPL1 and AMSH, and 40 nM for Cul1, Cul3, CSN2, and CSN5. A scrambled siRNA (Dharmacon) was used as a negative control. Knockdown cells were harvested 24 h after siRNA transfection or at times indicated in the figures. All siRNA sequences used in this study are listed in Supplementary Materials Table S1.

For overexpression of STAMBPL1 protein, AGS cells were transfected with 1 μg of pCMV-STAMBPL1 containing Myc-DDK tagged (OriGene) by using METAFECTENE^®^ PRO transfection reagent. Six hours after transfection, the medium was changed to fresh RPMI 1640 containing 10% FCS.

### Cell treatments

The cell culture medium was changed to fresh RPMI-1640 containing 10% FCS 4 h prior to the treatment of the cells. For infection, the *H. pylori* bacteria were suspended in phosphate-buffered saline (PBS) (Gibco^®^/Life Technologies) and eukaryotic cells were infected at a multiplicity of infection (MOI) of 50, 100 or 200 as indicated or stimulated with 10 ng/ml TNF or 10 ng/ml IL-1β (PeproTech). Camptothecin (CPT), doxorubicin (DOX) (Sigma) and staurosporine (STS) (Enzo Life Sciences GmbH) were used at the final concentration of 5 μM, 1 μM, and 1 μM, respectively. For cycloheximide (CHX) chase experiment, cells were pretreated with CHX (50 µg/ml) (Sigma) for 30 min prior to *H. pylori* infection. For ubiquitinylation analysis, MG132 (20 µM) (Selleckchem) or Lactacystin (10 µM) (Sigma) was added at times indicated in the figures. MLN4924 (Active Biochem) was used at the concentration of 1 µM.

### Preparation of whole cell lysates and subcellular fractionation

Cells were lysed for 30 min on ice in RIPA buffer (50 mM Tris/HCl pH 7.5, 150 mM NaCl, 2 mM EDTA, 10 mM K_2_HPO_4_, 10% glycerol, 1% Triton X-100, 0.05% SDS) supplemented with 1 mM AEBSF, 1 mM sodium vanadate, 1 mM sodium molybdate, 20 mM sodium fluoride, 20 mM 2-phosphoglycerate, and protease inhibitor cocktail (cOmplete^™^, Mini, EDTA-free, Roche). Afterwards, lysates were cleared by centrifugation (13,000 × g, 10 min, 4 °C). Furthermore, 7.5 mM N-ethylmaleimide (NEM) and 5 mM 1,10-phenantroline (OPT) (Sigma) were added to the lysis buffer.

Nuclear and cytoplasmic cell fractions were generated by subcellular fractionation as described previously [58]. Protein concentration was determined using the Pierce™ BCA protein assay kit (Thermo Fisher Scientific) according to the manufacturer’s instructions.

### SDS-PAGE and immunoblotting

Samples were mixed with Laemmli’s loading buffer, boiled for 5 min at 95 °C, separated by SDS-PAGE and electrotransferred onto PVDF membranes (Millipore) at 100 V constant. Membranes were blocked for 1 h at room temperature using 5% skim milk in TBS containing 0.1% Tween (TBS-T) and incubated with primary antibodies overnight in either 5% BSA or 5% skim milk in TBS-T at 4 °C on a rocking platform. The membranes were washed three times in TBS-T and incubated with the appropriate HRP-conjugated secondary antibody for 1 h at room temperature, followed by three washes in TBS-T. Immunoblots were developed using a chemiluminescent substrate (Millipore) and visualized using the ChemoCam Imager (Intas). All antibodies used in this study are listed in Supplementary Materials Table S2 and S3.

### Immunoprecipitation

Equal amounts of protein (0.5–1 mg in a volume of 700 μl) were incubated with 1 μg of specific antibody or with isotype IgG of the same species as a negative control (Sigma) overnight on a permanent rotator (7 rpm, 4 °C). Afterwards, pre-washed Pierce^™^ protein A/G magnetic beads (Thermo Fisher Scientific) were added to the reaction and rotated for 2 h at 4 °C. The beads were washed four times with RIPA buffer containing all inhibitors and eluted in 2 × Laemmli sample buffer (30 μl) for 20 min at room temperature. The eluate was transferred to a clean tube and heated for 5 min at 95 °C. Further, the IP buffer was additionally supplemented with 7.5 mM NEM and 5 mM OPT.

For immunoprecipitation (IP) of the CSN complex, pre-washed protein G sepharose beads (GE Healthcare) were added to the cell lysate in mono-detergent buffer (50 mM Tris/HCl pH 7.4, 150 mM NaCl, 1 mM EDTA, 1% Triton X-100, 1 × protease inhibitor cocktail) and allowed to proceed for 2 h at 4 °C. After centrifugation (500 × g, 2 min, 4 °C), supernatants were discarded, and beads were washed four times in 700 μl of IP mono-detergent buffer. After complete removal of the supernatants, beads were suspended in 30 μl 2 × Laemmli sample buffer.

IP buffer for in vitro binding assay contains 20 mM Tris/HCl pH 7.4, 150 mM NaCl, 2 mM EDTA, 1% Triton X-100, 0.1% SDS, 1 mM sodium vanadate, 20 mM sodium fluoride, and 1 × protease inhibitor cocktail. Pre-washed Pierce™ protein A/G magnetic beads were used (see above).

### RNA isolation, reverse transcription and quantitative PCR

Total RNA from AGS cells was extracted using the NucleoSpin^®^ RNA Plus kit (Macherey–Nagel) according to the manufacturer’s protocol. 1 μg of RNA was reverse transcribed into cDNA using RT^2^ First Strand Kit (Qiagen) according to the manual on the C1000 Thermal cycler (Bio-Rad). The quantitative PCR was performed on a StepOnePlus™ real time qPCR platform (Applied Biosystems) using the TaqMan^®^ Fast Universal PCR Master Mix and TaqMan^®^ assays specific for STAMBPL1 (Hs00697415_m1) and GAPDH (Hs99999905_m1) (Thermo Fisher Scientific). Relative quantification of gene expression was performed with the comparative CT method (ΔΔC_t_). RT-qPCR specificity was controlled by no-template and no-RT samples.

### Measurement of cellular ROS

The cellular ROS level was detected using the 2′,7′-dichlorodihydrofluorescein diacetate (H2DCF-DA), which can be oxidized to a highly fluorescent 2′,7′-dichlorofluorescein (DCF). AGS cells grown to confluence on 60 mm culture dish were treated with *H. pylori* (MOI 100 *wt*, *cagA*, *virB7*), chemotherapeutics (5 μM CPT, 1 μM DOX, 1 μM STS) or cytokines (10 ng/ml IL-1*β*, 10 ng/ml TNF) for 4 h. For the analysis of intracellular ROS levels, cells were stained with 20 μM H2DCF-DA for 30 min at 37 °C according to the manufacturer’s protocol and DCF fluorescence was measured by flow cytometry (CyFlow space). Hydrogen-peroxide (H_2_O_2_)-treated cells served as positive control and tert-butyl hydrogen peroxide (TBHP) (50 µM) was used as a positive control for analysis setting. Where indicated, AGS cells were treated with 20 or 40 μM of ROS inhibitor pyrrolidine dithiocarbamate (PDTC) (Selleckchem). Data were reported as the mean ± standard deviation of at least *n *= 3 different experiments. For DCF imaging, images were captured with a 20 × objective in green channel by the IncuCyte^®^ S3 Live Cell Analysis System (Sartorius, Essen Biosciences).

### In vitro translation and in vitro binding assay

CSN constructs were cloned as described previously [59]. Protein expression of CSN subunits was performed with the PURExpress^®^ In Vitro Protein Synthesis kit (New England Biolabs) according to the manufacturer’s protocol for 3 h at 37 °C. Final reaction volumes were 25 µl and contained 300 ng plasmid. Afterwards, equal molar amounts of recombinant human GST-STAMBPL1 (R&D System) were mixed and coincubated for 1 h at 37 °C, followed by IP using the respective anti-GST antibody. The IPs were separated by SDS-PAGE and analysed by immunoblots. Recombinant human GST protein (Abcam) was used as a negative control.

### In vitro DUB assay

AGS cells were transfected with siRNA against STAMBPL1 for 24 h, followed by MG132 treatment for 4 h before harvesting the cells. Survivin was immunoprecipitated from the STAMBPL1-depleted cells and eluted in an elution buffer (0.1 M glycine, pH 2.5) for 10 min at room temperature. The eluted fractions are neutralized immediately after recovery by addition of 1/10th volume of 1 M Tris–HCL, pH 8.5. In vitro DUB assay was carried out in 10 μl reactions containing 500 ng of ubiquitinylated immunoprecipitated Survivin and 100 nM of GST-tagged STAMBPL1 in 1 × ubiquitinylation assay buffer (50 mM HEPES pH 8.0, 100 mM NaCl, 1 mM DTT). The reactions were performed in the presence or absence of phenanthroline (5 mM) at 30 °C for 2 h and stopped by addition of 4 × Laemmli sample buffer. Samples were then separated by SDS-PAGE and analysed by immunoblots.

### Apoptotic cell death analysis by flow cytometry

Apoptotic cell death was determined by Annexin V/PI staining (MabTag GmbH). Briefly, 24 h after siRNA/cDNA transfection, cells were infected with *H. pylori* for 24 h. The cells were harvested using trypsin and stained with an Annexin V-FITC/PI Kit according to the manufacturer's instructions. The detection of apoptotic cell death was carried out on a flow cytometer (CyFlow space). Data were processed using Flowing Software 2 and the percentages of apoptotic cells were calculated.

### Caspase-3/7 assay

The IncuCyte^®^ Caspase-3/7 Reagents (Essen Bioscience) are a non-fluorescent membrane-permeable substrate, which can release a fluorescent DNA-intercalating dye when activated by executioner caspases, cleaved caspases-3/7, in the apoptotic pathway. After 24 h of siRNA/cDNA transfection, cells were re-seeded at a density of 50,000 cells/well in a 24-well plate and allowed to adhere overnight. Cell culture media were changed to media containing *H. pylori* (MOI 100) and IncuCyte^®^ Caspase-3/7 Green reagent diluted to the manufacturer’s recommended concentration. Plates were pre-warmed to 37 °C for 30 min before data acquisition to avoid condensation and expansion of the plate. Caspase-3/7 cleavage was measured every 2 h in an IncuCyte^®^ S3 Live Cell Analysis System (Sartorius, Essen Biosciences) and four image sets from distinct regions per well were captured with phase contrast and green channel at a magnification of 20 × . Analyses were performed by IncuCyte^®^ S3 Live Cell Analysis System integrated software and the percentages of apoptotic cells were calculated.

### Statistical analysis

All quantitative data were repeated at least two times and presented as mean ± S.D (standard deviation). Statistical analysis was performed using Student’s *t* test (SPSS Statistics 18.0). Values of *p* ≤ 0.05 and *p* ≤ 0.01 were considered as significant (*, **). N.S. stands for not statistically significant.

## Supplementary Information

Below is the link to the electronic supplementary material.

Supplementary file1 (PDF 2511 KB)

## Data Availability

All data generated and analyzed during the current study are included in this published article and its additional files.

## References

[1] Senft D, Qi J, Ronai ZA (2018). Ubiquitin ligases in oncogenic transformation and cancer therapy. Nat Rev Cancer.

[2] Duda DM, Borg LA, Scott DC, Hunt HW, Hammel M, Schulman BA (2008). Structural insights into NEDD8 activation of cullin-RING ligases: conformational control of conjugation. Cell.

[3] Enchev RI, Schulman BA, Peter M (2015). Protein neddylation: beyond cullin-RING ligases. Nat Rev Mol Cell Biol.

[4] Clague MJ, Urbé S, Komander D (2019). Breaking the chains: deubiquitylating enzyme specificity begets function. Nat Rev Mol Cell Biol.

[5] Mevissen TET, Komander D (2017). Mechanisms of deubiquitinase specificity and regulation. Annu Rev Biochem.

[6] Guo Y, Liu Q, Mallette E, Caba C, Hou F, Fux J (2021). Structural and functional characterization of ubiquitin variant inhibitors for the JAMM-family deubiquitinases STAMBP and STAMBPL1. J Biol Chem.

[7] Ambroise G, Yu TT, Zhang B, Kacal M, Hao Y, Queiroz AL (2020). Systematic analysis reveals a functional role for STAMBPL1 in the epithelial-mesenchymal transition process across multiple carcinomas. Br J Cancer.

[8] Kikuchi K, Ishii N, Asao H, Sugamura K (2003). Identification of AMSH-LP containing a Jab1/MPN domain metalloenzyme motif. Biochem Biophys Res Commun.

[9] Ribeiro-Rodrigues TM, Catarino S, Marques C, Ferreira JV, Martins-Marques T, Pereira P (2014). AMSH-mediated deubiquitination of Cx43 regulates internalization and degradation of gap junctions. FASEB J.

[10] Chen X, Shi H, Bi X, Li Y, Huang Z (2019). Targeting the deubiquitinase STAMBPL1 triggers apoptosis in prostate cancer cells by promoting XIAP degradation. Cancer Lett.

[11] Yu DJ, Qian J, Jin X, Li J, Guo CX, Yue XC (2019). STAMBPL1 knockdown has antitumour effects on gastric cancer biological activities. Oncol Lett.

[12] Lavorgna A, Harhaj EW (2012). An RNA interference screen identifies the deubiquitinase STAMBPL1 as a critical regulator of human T-cell leukemia virus type 1 tax nuclear export and NF-κB activation. J Virol.

[13] Srinivas US, Tan BWQ, Vellayappan BA, Jeyasekharan AD (2019). ROS and the DNA damage response in cancer. Redox Biol.

[14] Snyder NA, Silva GM (2021). Deubiquitinating enzymes (DUBs): regulation, homeostasis, and oxidative stress response. J Biol Chem.

[15] Ma Y, Zhang L, Rong S, Qu H, Zhang Y, Chang D (2013). Relation between gastric cancer and protein oxidation, DNA damage, and lipid peroxidation. Oxid Med Cell Longev.

[16] Moss SF, Calam J, Agarwal B, Wang S, Holt PR (1996). Induction of gastric epithelial apoptosis by *Helicobacter pylori*. Gut.

[17] Peek RM, Moss SF, Tham KT, Pérez-Pérez GI, Wang S, Miller GG (1997). *Helicobacter pylori* cagA+ strains and dissociation of gastric epithelial cell proliferation from apoptosis. J Natl Cancer Inst.

[18] Bhattacharyya A, Chattopadhyay R, Burnette BR, Cross JV, Mitra S, Ernst PB (2009). Acetylation of apurinic/apyrimidinic endonuclease-1 regulates Helicobacter pylori-mediated gastric epithelial cell apoptosis. Gastroenterology.

[19] Liu JF, Guo D, Kang EM, Wang YS, Gao XZ, Cong HY (2021). Acute and chronic infection of *H. pylori* caused the difference in apoptosis of gastric epithelial cells. Microb Pathog.

[20] Brahma MK, Gilglioni EH, Zhou L, Trépo E, Chen P, Gurzov EN (2021). Oxidative stress in obesity-associated hepatocellular carcinoma: sources, signaling and therapeutic challenges. Oncogene.

[21] Woo SM, Seo SU, Kubatka P, Min KJ, Kwon TK (2019). Honokiol enhances TRAIL-mediated apoptosis through STAMBPL1-induced Survivin and c-FLIP degradation. Biomolecules.

[22] Liu Y, Lear T, Iannone O, Shiva S, Corey C, Rajbhandari S (2015). The proapoptotic F-box protein Fbxl7 regulates mitochondrial function by mediating the ubiquitylation and proteasomal degradation of Survivin. J Biol Chem.

[23] Kamran M, Long ZJ, Xu D, Lv SS, Liu B, Wang CL (2017). Aurora kinase A regulates Survivin stability through targeting FBXL7 in gastric cancer drug resistance and prognosis. Oncogenesis.

[24] Arora V, Cheung HH, Plenchette S, Micali OC, Liston P, Korneluk RG (2007). Degradation of survivin by the X-linked inhibitor of apoptosis (XIAP)-XAF1 complex. J Biol Chem.

[25] Ding SZ, Minohara Y, Fan XJ, Wang J, Reyes VE, Patel J (2007). *Helicobacter pylori* infection induces oxidative stress and programmed cell death in human gastric epithelial cells. Infect Immun.

[26] Yang H, Villani RM, Wang H, Simpson MJ, Roberts MS, Tang M (2018). The role of cellular reactive oxygen species in cancer chemotherapy. J Exp Clin Cancer Res.

[27] Loignon M, Miao W, Hu L, Bier A, Bismar TA, Scrivens PJ (2009). Cul3 overexpression depletes Nrf2 in breast cancer and is associated with sensitivity to carcinogens, to oxidative stress, and to chemotherapy. Mol Cancer Ther.

[28] Bramasole L, Sinha A, Gurevich S, Radzinski M, Klein Y, Panat N (2019). Proteasome lid bridges mitochondrial stress with Cdc53/Cullin1 NEDDylation status. Redox Biol.

[29] Cope GA, Suh GS, Aravind L, Schwarz SE, Zipursky SL, Koonin EV (2002). Role of predicted metalloprotease motif of Jab1/Csn5 in cleavage of Nedd8 from Cul1. Science.

[30] Schwechheimer C, Serino G, Deng XW (2002). Multiple ubiquitin ligase-mediated processes require COP9 signalosome and AXR1 function. Plant Cell.

[31] Naumann M, Bech-Otschir D, Huang X, Ferrell K, Dubiel W (1999). COP9 signalosome-directed c-Jun activation/stabilization is independent of JNK. J Biol Chem.

[32] Leppert U, Henke W, Huang X, Müller JM, Dubiel W (2011). Post-transcriptional fine-tuning of COP9 signalosome subunit biosynthesis is regulated by the c-Myc/Lin28B/let-7 pathway. J Mol Biol.

[33] Dubiel W, Chaithongyot S, Dubiel D, Naumann M (2020). The COP9 signalosome: a multi-DUB complex. Biomolecules.

[34] Huang X, Ordemann J, Pratschke J, Dubiel W (2016). Overexpression of COP9 signalosome subunits, CSN7A and CSN7B, exerts different effects on adipogenic differentiation. FEBS Open Bio.

[35] Sies H, Jones DP (2020). Reactive oxygen species (ROS) as pleiotropic physiological signalling agents. Nat Rev Mol Cell Biol.

[36] Shang F, Taylor A (2011). Ubiquitin-proteasome pathway and cellular responses to oxidative stress. Free Radic Biol Med.

[37] Liang J, Cao R, Wang X, Zhang Y, Wang P, Gao H (2017). Mitochondrial PKM2 regulates oxidative stress-induced apoptosis by stabilizing Bcl2. Cell Res.

[38] Harper JW, Schulman BA (2021). Cullin-RING ubiquitin ligase regulatory circuits: a quarter century beyond the F-box hypothesis. Annu Rev Biochem.

[39] Huang X, Dubiel D, Dubiel W (2021). The COP9 signalosome variant CSNCSN7A stabilizes the deubiquitylating enzyme CYLD impeding hepatic steatosis. Livers.

[40] Schweitzer K, Bozko PM, Dubiel W, Naumann M (2007). CSN controls NF-kappaB by deubiquitinylation of IkappaBalpha. EMBO J.

[41] Schweitzer K, Naumann M (2015). CSN-associated USP48 confers stability to nuclear NF-κB/RelA by trimming K48-linked Ub-chains. Biochim Biophys Acta.

[42] Tomoda K, Kubota Y, Arata Y, Mori S, Maeda M, Tanaka T (2002). The cytoplasmic shuttling and subsequent degradation of p27Kip1 mediated by Jab1/CSN5 and the COP9 signalosome complex. J Biol Chem.

[43] Yun J, Tomida A, Andoh T, Tsuruo T (2004). Interaction between glucose-regulated destruction domain of DNA topoisomerase IIalpha and MPN domain of Jab1/CSN5. J Biol Chem.

[44] Park YH, Jeong MS, Ha KT, Yu HS, Jang SB (2017). Structural characterization of As-MIF and hJAB1 during the inhibition of cell-cycle regulation. BMB Rep.

[45] Zhou R, Shao Z, Liu J, Zhan W, Gao Q, Pan Z (2018). COPS5 and LASP1 synergistically interact to downregulate 14-3-3σ expression and promote colorectal cancer progression via activating PI3K/AKT pathway. Int J Cancer.

[46] Manohar S, Jacob S, Wang J, Wiechecki KA, Koh HWL, Simões V (2019). Polyubiquitin chains linked by lysine residue 48 (K48) selectively target oxidized proteins in vivo. Antioxid Redox Signal.

[47] Naumann M, Sokolova O, Tegtmeyer N, Backert S (2017). *Helicobacter pylori*: A paradigm pathogen for subverting host cell signal transmission. Trends Microbiol.

[48] Saha A, Backert S, Hammond CE, Gooz M, Smolka AJ (2010). *Helicobacter pylori* CagL activates ADAM17 to induce repression of the gastric H, K-ATPase alpha subunit. Gastroenterology.

[49] Sokolova O, Vieth M, Gnad T, Bozko PM, Naumann M (2014). *Helicobacter pylori* promotes eukaryotic protein translation by activating phosphatidylinositol 3 kinase/mTOR. Int J Biochem Cell Biol.

[50] Cui X, Shen D, Kong C, Zhang Z, Zeng Y, Lin X (2017). NF-κB suppresses apoptosis and promotes bladder cancer cell proliferation by upregulating survivin expression in vitro and in vivo. Sci Rep.

[51] Maubach G, Lim MCC, Sokolova O, Backert S, Meyer TF, Naumann M (2021). TIFA has dual functions in *Helicobacter pylori*-induced classical and alternative NF-κB pathways. EMBO Rep.

[52] Lim MCC, Maubach G, Sokolova O, Feige MH, Diezko R, Buchbinder J (2017). Pathogen-induced ubiquitin-editing enzyme A20 bifunctionally shuts off NF-κB and caspase-8-dependent apoptotic cell death. Cell Death Differ.

[53] Xu H, Yu J, Cui J, Chen Z, Zhang X, Zou Y (2021). Ablation of Survivin in T cells attenuates acute allograft rejection after murine heterotopic heart transplantation by inducing apoptosis. Front Immunol.

[54] Pavlyukov MS, Antipova NV, Balashova MV, Vinogradova TV, Kopantzev EP, Shakhparonov MI (2011). Survivin monomer plays an essential role in apoptosis regulation. J Biol Chem.

[55] Zhou J, Guo X, Chen W, Wang L, Jin Y (2020). Targeting survivin sensitizes cervical cancer cells to radiation treatment. Bioengineered.

[56] Marusawa H, Matsuzawa S, Welsh K, Zou H, Armstrong R, Tamm I (2003). HBXIP functions as a cofactor of survivin in apoptosis suppression. EMBO J.

[57] Song Z, Liu S, He H, Hoti N, Wang Y, Feng S (2004). A single amino acid change (Asp 53 → Ala53) converts Survivin from anti-apoptotic to pro-apoptotic. Mol Biol Cell.

[58] Studencka-Turski M, Maubach G, Feige MH, Naumann M (2018). Constitutive activation of nuclear factor kappa B-inducing kinase counteracts apoptosis in cells with rearranged mixed lineage leukemia gene. Leukemia.

[59] Lee JH, Yi L, Li J, Schweitzer K, Borgmann M, Naumann M (2013). Crystal structure and versatile functional roles of the COP9 signalosome subunit 1. Proc Natl Acad Sci USA.

