# Supplementary material for: Helicobacter pylori-induced reactive oxygen species direct turnover of CSN-associated STAMBPL1 and augment apoptotic cell death
Source: Cell Mol Life Sci. 2022 Jan 23;79(2):86. doi: 10.1007/s00018-022-04135-2 (PMC8784504; doi:10.1007/s00018-022-04135-2)

“*Helicobacter pylori*-induced reactive oxygen species direct turnover of CSN-associated STAMBPL1 and augments apoptotic cell death”

Supattra Chaithongyot , Michael Naumann

Supplementary Data

Supplementary Fig. S1

(a) Sequence alignment of STAMBPL1 and AMSH. Alignment was performed with Clustal X. The underline shows the MPN domain. Identical amino acids are marked with asterisks. (b) NCI-N87, (c) HeLa, and (d) NCI-N87 polarised cells were infected with *H. pylori* P1wt at MOI 100 for the indicated time points. STAMBPL1 protein level was analyzed by IB.

Supplementary Fig. S2

(a) HeLa cells were infected with *H. pylori* (a) P1wt or (b) V*irB7* in the presence or absence of MLN4924 (1 μM), followed by IB analysis of STAMBPL1.

Supplementary Fig. S3

Equal molar amounts of *in vitro*-translated Flag-CSN and recombinant His-USP15 were incubated at 37 °C for 1 h followed by IP with an anti-His antibody.

Supplementary Fig. S4

AGS cells were transfected with siRNA-2 against STAMBPL1 for 24 h and then infected with *H. pylori* for 24 h. Survivin and caspase-3 cleavage were analysed by IB.

Supplementary Figure 1

S1a (To Fig.1a)

```
AMSH           1 MSDHGDVSLPPEDRVRALSQLGSAVEVNEDIPPRRYFRSGVEIIRMASIYSEEGNIEHAF
STAMBPL1      16 MPDHTDVSLSPEERVRALSKLGCNITISEDITPRRYFRSGVEMERMASVYLEEGNLENAF
                 * ** **** ** ****** **      *** ********** **** * **** * **

AMSH          61 ILYNKYITLFIEKLPKHRDYKSAVIPEKKDTVKKLKEIAFPKAEELKAELLKRYTKEYTE
STAMBPL1      76 VLYNKFITLFVEKLPNHRDYQQCAVPEKQDIMKKLKEIAFPRTDELKNDLLKKYNVEYQE
                  **** **** **** ****     *** *  *********   ***  *** *  ** *

AMSH         121 YNEEKKKEAEELARNMAIQQELEKEKQRVAQQKQQQLEQEQFHAFEEMIRNQELEKERLK
STAMBPL1     136 YLQSKNKYKAEILKKLEHQRLIEAERKRIAQMRQQQLESEQFLFFEDQLKKQELARGQMR
                 *   * *   *       *   * *  * **  ***** ***  **     ***

AMSH         181 IVQEFGKVDPGLGGPLVPDLEKPSLDVFPTLTVSSIQPSDCHTTVRPAKPPVVDRSLKPG
STAMBPL1     196 SQQTSGLSEQIDGSALSCFSTHQNNSL---LNVFADQPNKSDATNYASHSPPVNRALTPA
                   *  *      *  *              * *   **     *      * * * * *

AMSH         241 ALSNSESIPTIDGLRHVVVPGRLCPQFLQLASANTARGVETCGILCGKLMRNEFTITHVL
STAMBPL1     253 ATLSAVQNLVVEGLRCVVLPEDLCHKFLQLAESNTVRGIETCGILCGKLTHNEFTITHVI
                 *           *** ** *  **  *****  ** ** **********  ********

AMSH         301 IPKQSAGSDYCNTENEEELFLIQDQQGLITLGWIHTHPTQTAFLSSVDLHTHCSYQMMLP
STAMBPL1     313 VPKQSAGPDYCDMENVEELFNVQDQHDLLTLGWIHTHPTQTAFLSSVDLHTHCSYQLMLP
                  ****** ***  ** ****  ***  * *************************** ***

AMSH         361 ESVAIVCSPKFQETGFFKLTDHGLEEISSCRQKGFHPHSKDPPLFCSCSHVTVVDRAVTI
STAMBPL1     373 EAIAIVCSPKHKDTGIFRLTNAGMLEVSACKKKGFHPHTKEPRLFSICKHVLVKDIKIIV
                 *  *******   ** * **  *  * * *  ****** * * **  * ** * *

AMSH         421 TDLR
STAMBPL1     433 LDLR
                  ***
```

S1b (To Fig.1b)

NCI-N87

P1wt [h] - 1 2 4 6 10 12 16 24 kDa

STAMBPL1

GAPDH

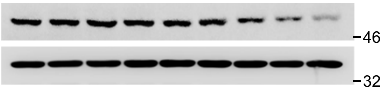

S1c (To Fig.1b)

HeLa

P1wt [h] - 1 2 4 6 10 12 16 24 kDa

STAMBPL1

GAPDH

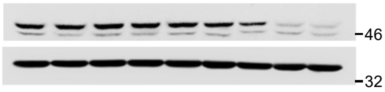

S1d (To Fig.1b)

NCI-N87 polarized cells

P1wt [h] - 9 12 16 20 24 kDa

STAMBPL1

GAPDH

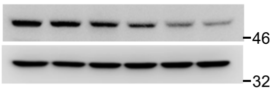

Supplementary Figure 2

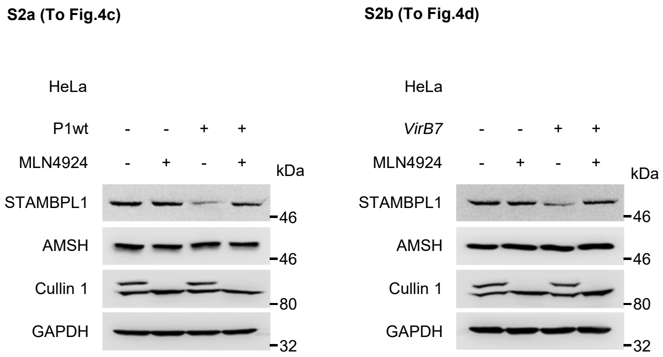

Supplementary Figure 3

S3 (To Fig.5f)

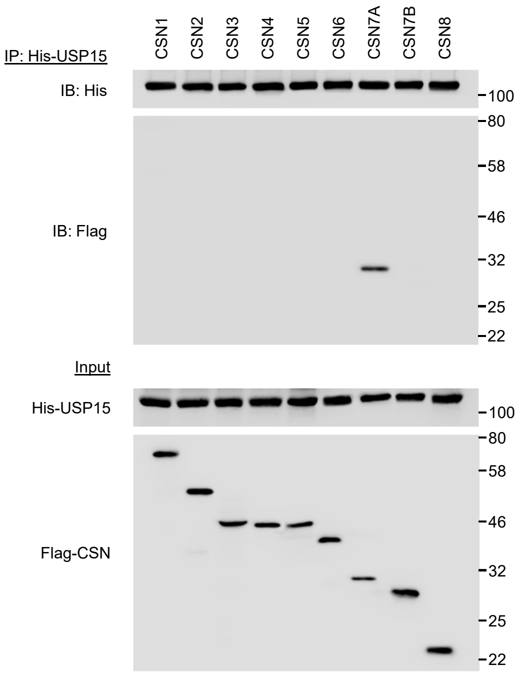

Supplementary Figure 4

S4 (To Fig.7a)

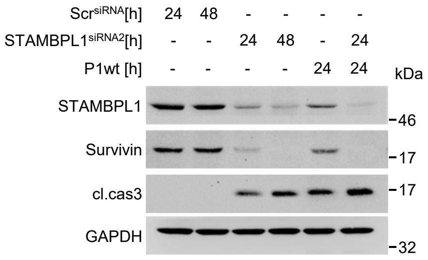

Supplementary Materials

Table S1 siRNAs used in the study

Target	siRNA sequence (5'->3')	Supplier	Catalogue number
STAMBPL1	CGUAGAAUACCAAGAAUAU	Eurofins Genomics	custom synthesized
AMSH	UUACAAAUCUGCUGUCAUUUU	Eurofins Genomics	custom synthesized
Cullin1	CAACGAAGAGUUCAGGUUU	Dharmacon	J-004086-06-0005
Cullin3	GAGAUCAAGUUGUACGUUA	Eurofins Genomics	custom synthesized
CSN2	AssayID: 140004	Thermo Fisher Scientific	AM16708
CSN5	AssayID: 108110	Thermo Fisher Scientific	AM16708

Table S2 Primary antibodies used in the study

Antibody	Supplier	Catalogue number
STAMBPL1	Santa Cruz Biotechnology	sc-376526
AMSH	Santa Cruz Biotechnology	sc-271641
Survivin	Santa Cruz Biotechnology	sc-17779
Cleaved caspase3	Cell Signaling Technology	#9661
Cullin1	Abcam	ab2964-500
Cullin3	Novus	NB100-58787
CSN2	Abcam	ab155774
CSN5	GeneTex	GTX70207
CSN6	Santa Cruz Biotechnology	sc-137153
CSN7A	Santa Cruz Biotechnology	sc-398882
Flagellin	Acris	AM00865PU-N
CagA	Austral Biologicals	#HPM-5001-5
GAPDH	Millipore	#MAB374
Nucleolin	Santa Cruz Biotechnology	sc-13057
Lamin B2	Santa Cruz Biotechnology	sc-377379
FLAG	Sigma-Aldrich	#F3165
DDDDK Tag	abcam	ab1162
GST	Santa Cruz Biotechnology	sc-53909
c-Myc	Santa Cruz Biotechnology	sc-40
c-Myc	Santa Cruz Biotechnology	sc-789
His	Santa Cruz Biotechnology	sc-53073
Ub (PAN)	Santa Cruz Biotechnology	sc-8017
Ubiquitin K48 linkage	Millipore	05-1307
USP15	Abnova	H00009958-M01
USP7	Bethyl	A300-033A

Table S3 Secondary antibodies used in the study

Antibody	Supplier	Catalogue number
Anti-mouse IgG-HRP	Santa Cruz Biotechnology	sc-2005
Anti-rabbit IgG-HRP	Santa Cruz Biotechnology	sc-2004
Anti-mouse IgG (Light chain)	Jackson ImmunoResearch	115-035-174
Anti-rabbit IgG (Light chain)	Jackson ImmunoResearch	211-032-171

Figure 1 uncropped immunoblots

b

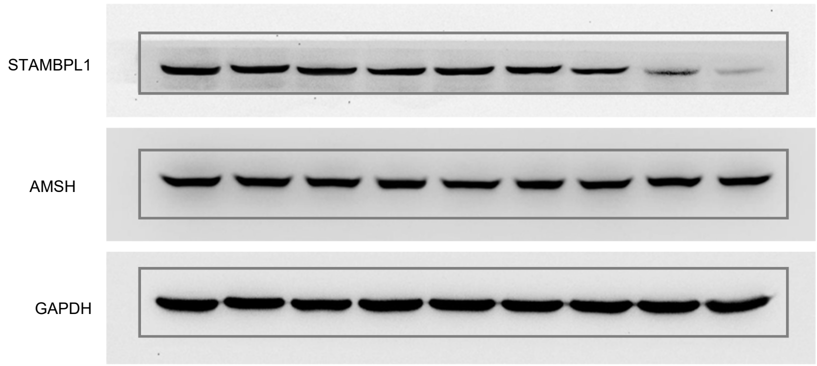

c

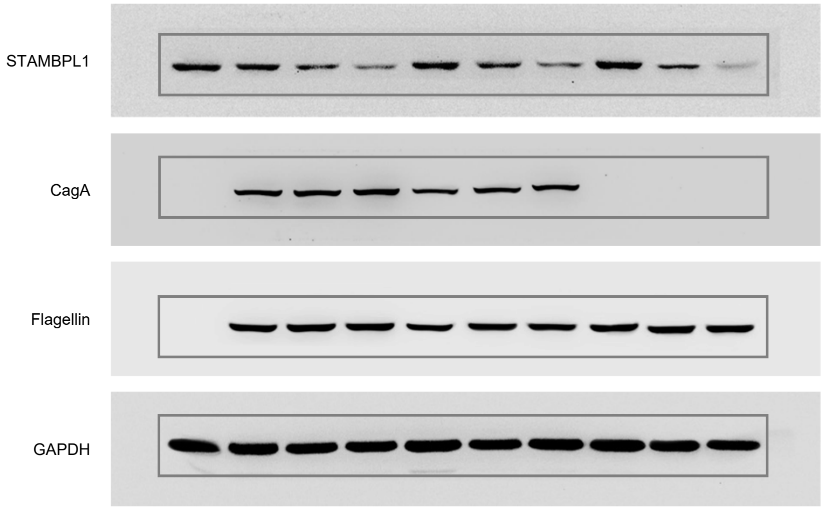

d

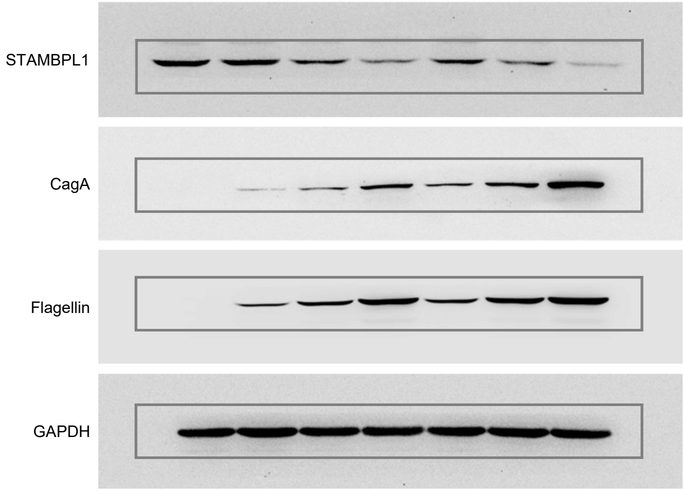

e

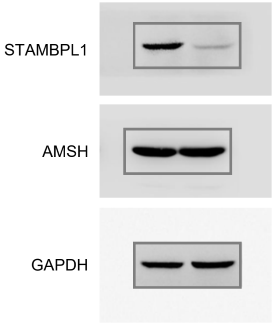

Figure 2 uncropped immunoblots

a

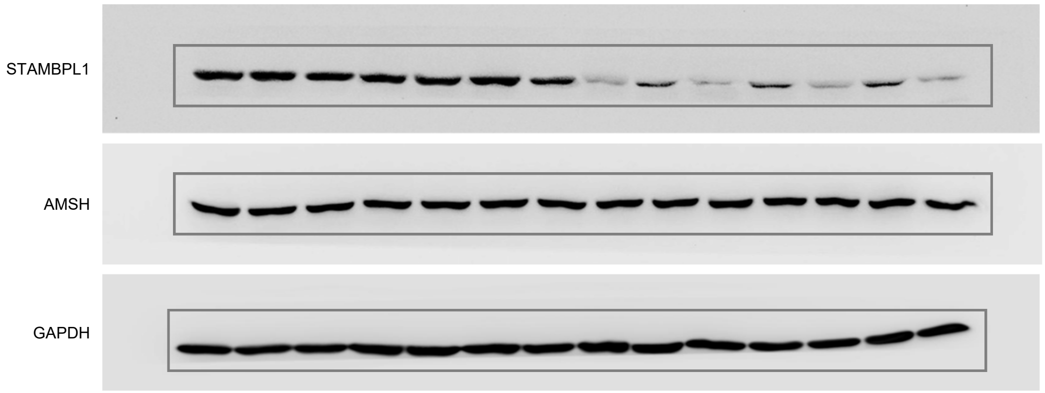

d

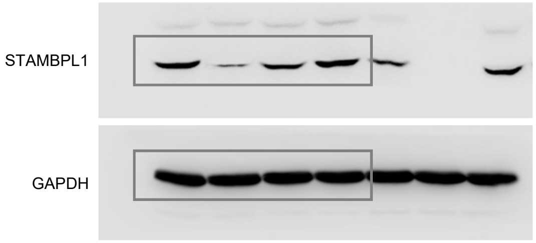

e

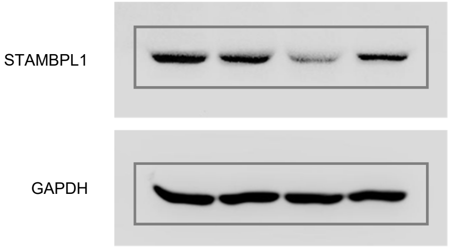

Figure 3 uncropped immunoblots

b

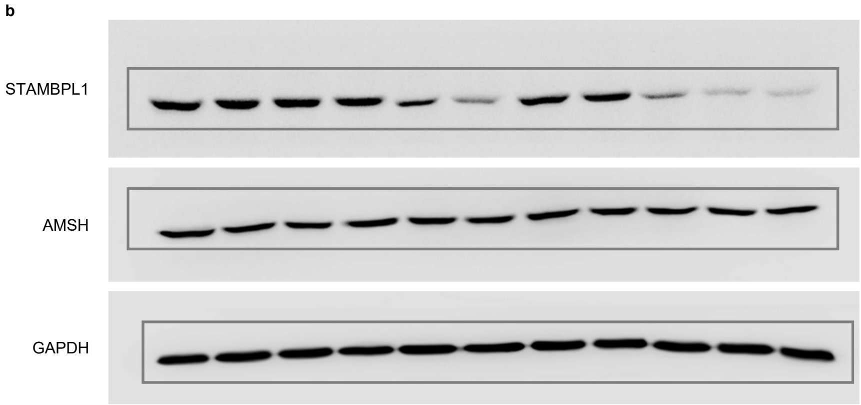

c

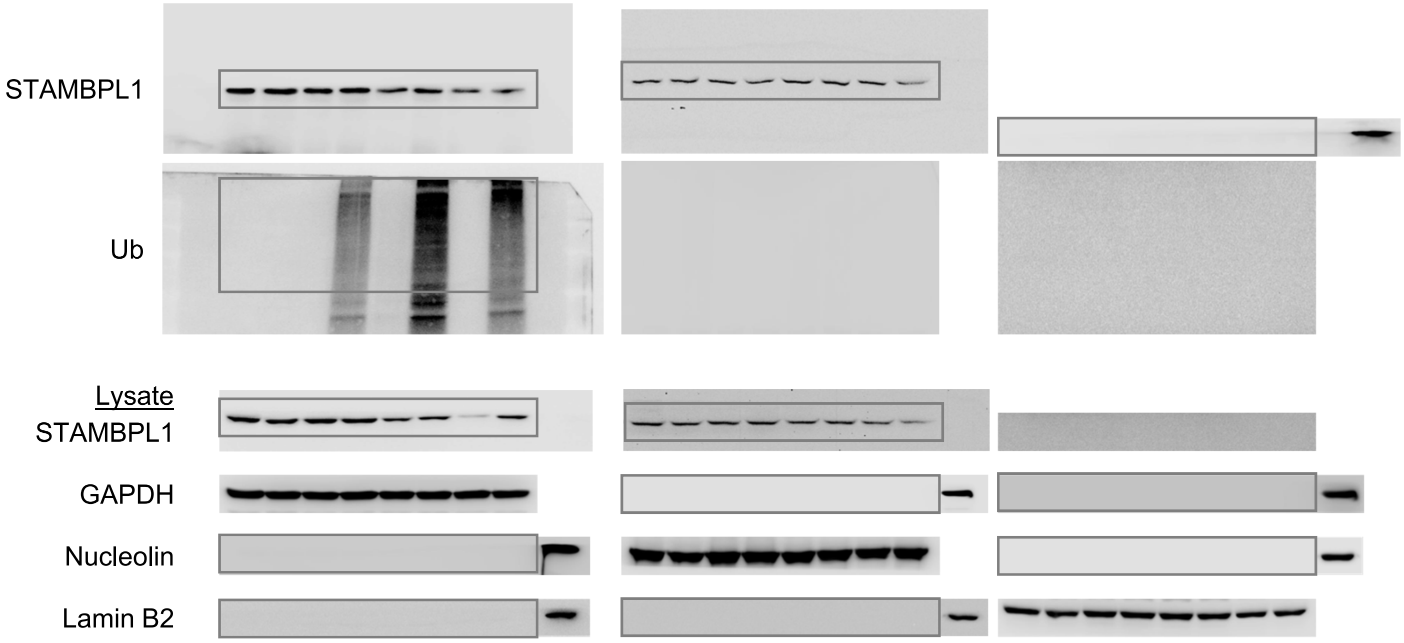

Figure 4 uncropped immunoblots

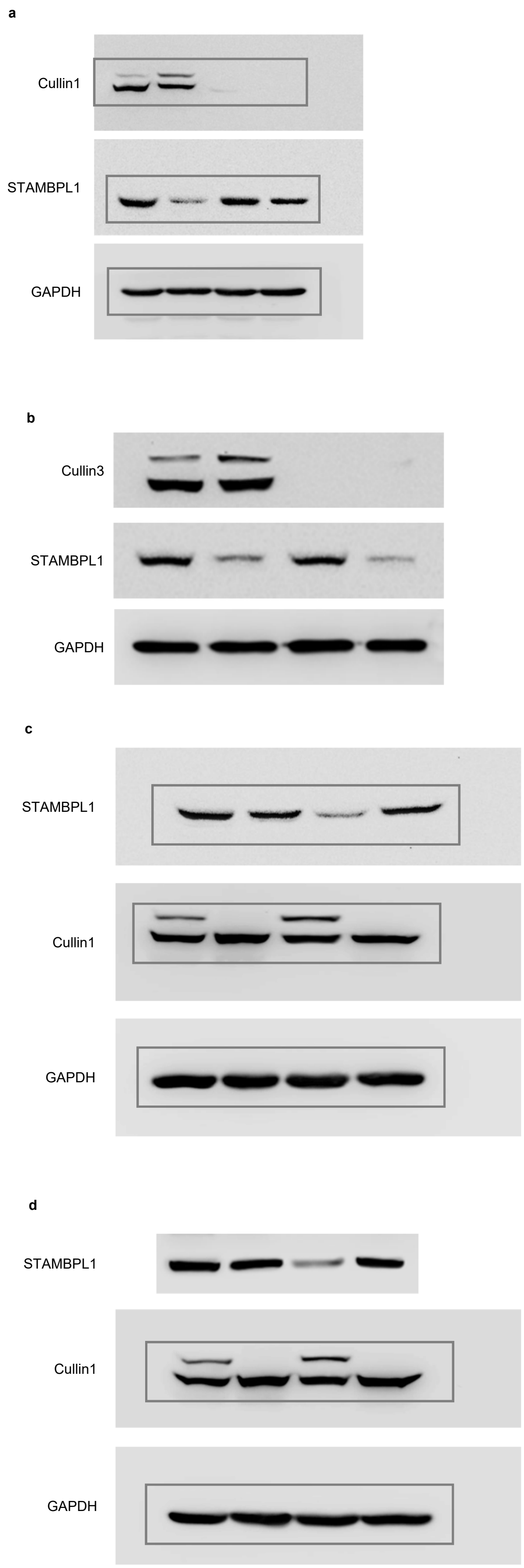

Figure 5 uncropped immunoblots

b

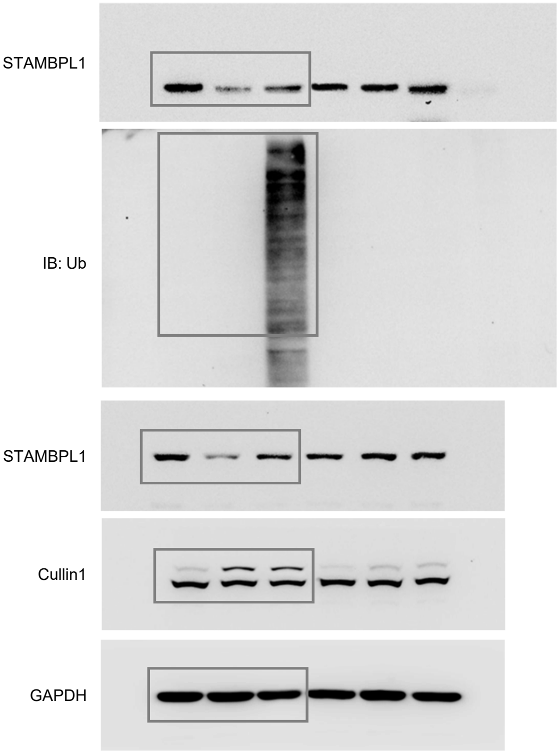

a

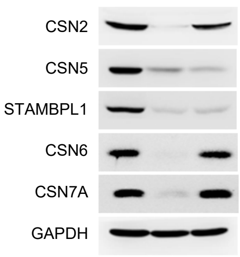

c

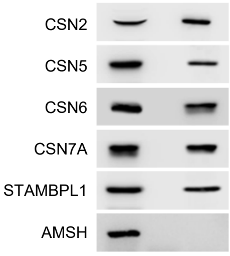

d

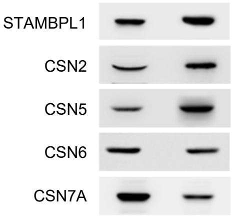

e

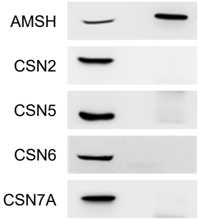

f

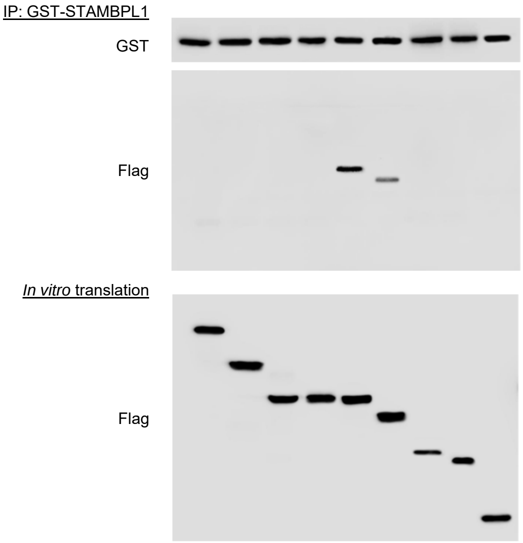

g

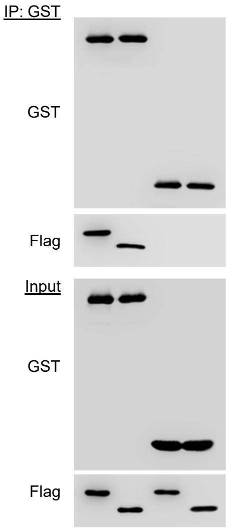

Figure 6 uncropped immunoblots

a

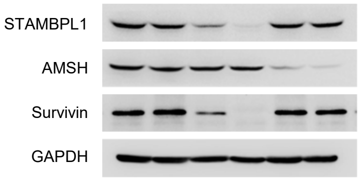

b

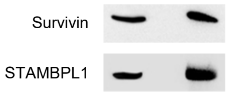

c

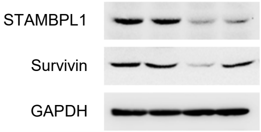

d

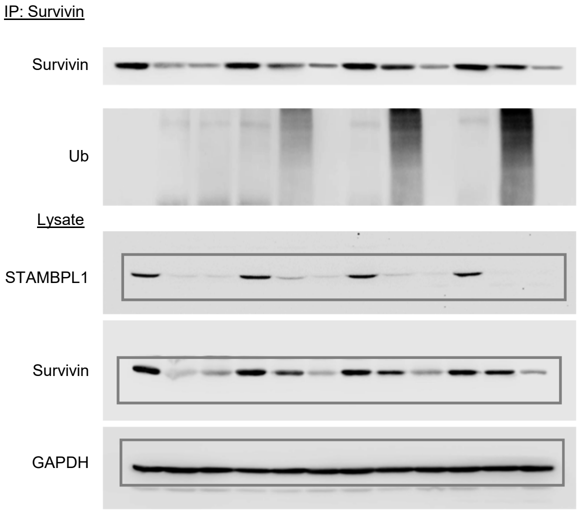

e

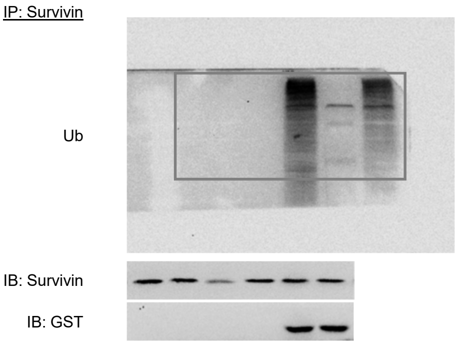

f

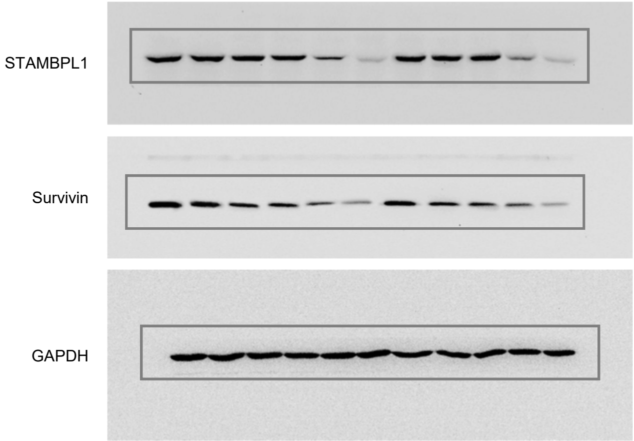

g

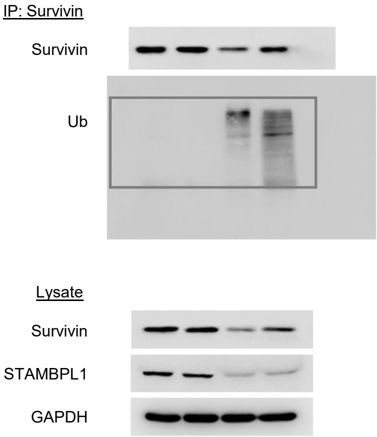

Figure 7 uncropped immunoblots

a

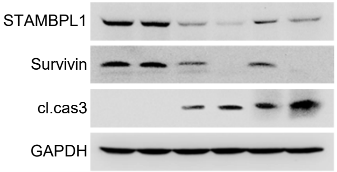

b

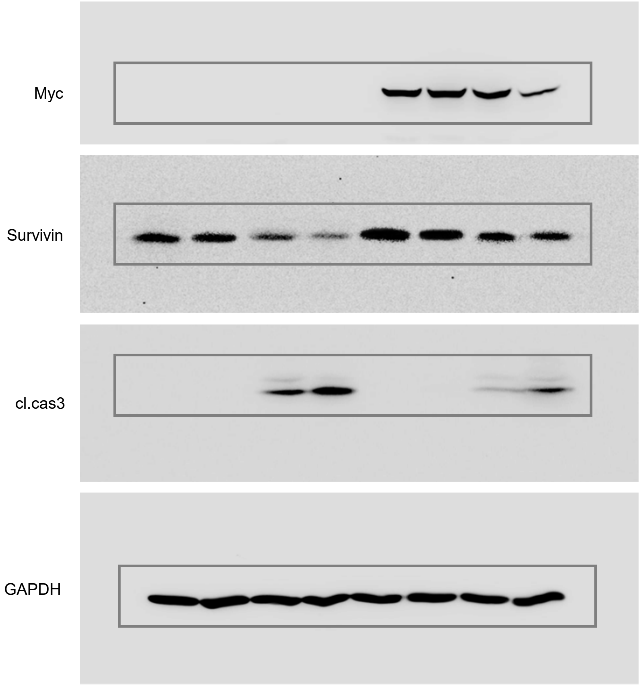

Figure S1 uncropped immunoblots

S1b

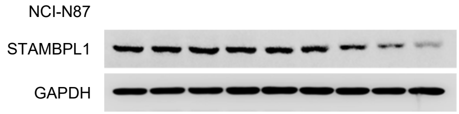

S1c

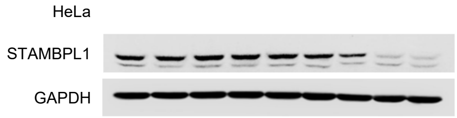

S1d

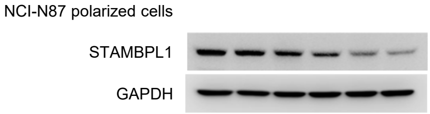

Figure S2 uncropped immunoblots

S2a

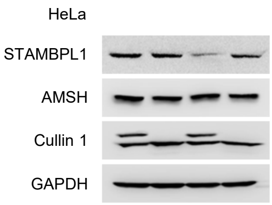

S2b

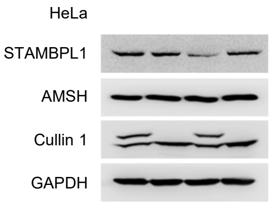

Figure S3 uncropped immunoblots

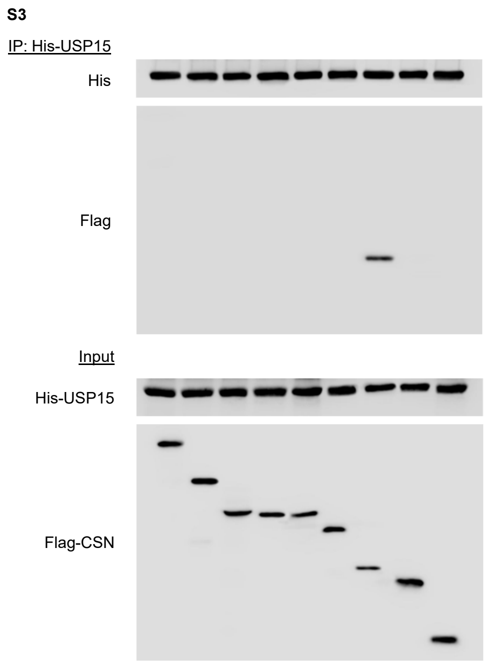

Figure S4 uncropped immunoblots

S4

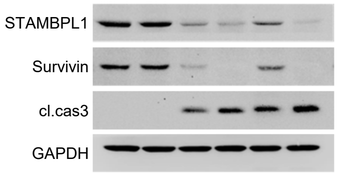

Supplement: Supplementary file 1 — Supplementary file1 (PDF 2511 KB) [file 18_2022_4135_MOESM1_ESM.pdf]
